# Tri-system integration in metal-oxide nanocomposites via in-situ solution-processed method for ultrathin flexible transparent electrodes

**DOI:** 10.1038/s41467-024-46243-6

**Published:** 2024-03-07

**Authors:** John Jinwook Kim, Kojima Shuji, Jiawei Zheng, Xinjun He, Ahmad Sajjad, Hong Zhang, Haibin Su, Wallace C. H. Choy

**Affiliations:** 1 Department of Electrical and Electronic Engineering, The University of Hong Kong, Pokfulam Road, Hong Kong, China; 2 State Key Laboratory of Photovoltaic Science and Technology, Shanghai Frontiers Science Research Base of Intelligent Optoelectronics and Perception, Institute of Optoelectronics, Fudan University, Shanghai, 200433 China; 3 Department of Chemistry, The Hong Kong University of Science and Technology, Clear Water Bay, Kowloon, Hong Kong, China

**Keywords:** Electronic devices, Electrical and electronic engineering, Electronic devices, Nanoparticles, Nanowires

## Abstract

For stable operation of ultrathin flexible transparent electrodes (uFTEs), it is critical to implement effective risk management during concurrent multi-loading operation of electrical bias and mechanical folding cycles in high-humidity environments. Despite extensive efforts in preparing solution-processed uFTEs with cost-effective and high-throughput means, achieving in-situ nano-adhesion in heterogeneous metal-oxide nanocomposites remains challenging. In this work, we observed by serendipity liquid-like behaviour of transparent metal-oxide-semiconductor zinc oxide nanoparticles (^ZnO^NPs) onto silver nanowires (^Ag^NWs) developed by in-situ solution processed method (iSPM). This enabled us to address the long-standing issue of vulnerability in the nanocomposite caused by the interface of dissimilar materials between ^Ag^NWs and ^ZnO^NPs, resulting in a remarkably improved multi-loading operation. Importantly, substrate-integrated uFTEs constituted of the metal-oxide nanocomposite electrode semi-embedded in the polymer matrix of greatly thin <0.5 μm thickness is successfully demonstrated with the smooth surface topography, promoted by the tri-system integration including (i) ^Ag^NW-^Ag^NW, (ii) ^ZnO^NP-^ZnO^NP, and (iii) ^Ag^NW-^ZnO^NP systems. Our finding unveils the complex interfacial dynamics associated with the heterogeneous interface system between ^Ag^NWs and ^ZnO^NPs and holds great promise in understanding the in-situ nano-adhesion process and increasing the design flexibility of next generation solution-processed uFTEs.

## Abstract (web summary)

Fabricating flexible transparent electrodes with robust operational stability alongside electrical and mechanical properties is challenging. Here, Kim et al. develop ultrathin flexible transparent electrodes using in-situ solution-processing and unveil interface dynamics of the integrated tri-system.

## Introduction

Recent advancements in reducing device thickness in the field of ultrathin flexible transparent electrodes (*u*FTEs) have been favorably prevailing in state-of-art research fields and modern device innovations such as wearable optoelectronic devices, implantable bioelectronics, soft robotics, aircraft electronics, and imperceptible electronic skin since a high level of compatibility and comfortability^1–6^. Previously, sputtered indium tin oxide embossed electrodes have been demonstrated on target surfaces of as-prepared ultrathin flexible substrates^7^. However, there is an ever-increasing demand for advanced solution-processed *u*FTEs with fast, facile, low-cost, and high-throughput printing production^8–10^. The stringent requirements include a high figure of merit with well-balanced electrical and optical properties (i.e. <10 Ω sq.^−1^ of sheet resistance and >90 % of diffused transmittance) and very smooth surface morphology (i.e. <1 nm of root-mean-square (*RMS*)) is preferred in terms of device applications^11–18^. More importantly, the high operational stability under simultaneous multi-loading operations (i.e. folding cycles with a few mm radius of curvature, current bias with several hundred mA cm^−2^ including efficient thermal energy management, and humid environments at >85 % of relative humidity) are highly desirable, which is one of the rigorous operational situations in real applications.

Among various solution-processable conductor approaches^10^, substrate-integrated *u*FTEs constituted of nanonets of silver nanowires (^*Ag*^NWs) embedded in flexible substrates have been demonstrated as the novel *u*FTE architectures with outstanding electrical/optical properties and mechanical flexibility by the means of fully solution-process^19^. However, the completely buried Ag nanonets in the insulating polymer matrix only allow narrow conductive pathways exposed to the top surface, which significantly limits the compatibility with various applications. Recently, there was a trial to extend the conductive pathways to adjacent layers by employing conductive polymers such as poly(3,4-ethylenedioxythiophene) polystyrene sulfonate in Ag nanonets for photovoltaic device applications^20^. However the severe instability issue increasing the resistance was observed due to the hygroscopic property of the conductive polymer in humid conditions. Meanwhile, Ag nanonets have been frequently combined with transparent metal-oxide-semiconductor nanomaterials to overcome not only the issue of the restricted conductive pathways but also avoid the vulnerability to externalities such as high humidity^21–24^. Nonetheless, there is still a big difficulty in achieving satisfactory performance against different operational tests. For instance, the Ag nanonets protected by atomically deposited transparent metal-oxide-semiconductors such as zinc oxide (ZnO) will break down under a continuous bias with a current density above 155 mA cm^−2^ due to strong Gibbs-Thomson effects in Joule heating to the Ag nanonets^25^. Besides, although the superior operation of ZnO-Ag nanonets under 5000-cycled bending has been reported with 3 mm of a bending radius^26^, there are many cracks appeared between Ag nanonets and the adjacent ZnO matrix in ZnO-Ag nanonets embedded in the polymer matrix with >20 nm of peak-to-valley (*PtV*) surface roughness, which will unfavorably accelerate the formation of mechanical defects under the repetitive mechanical bendings. Furthermore, there are very few studies on the high operational stability of *u*FTEs applicable to practical multi-loading fatigue examinations.

One of resolving the fundamental bottleneck of the instability issue is to increase adhesive interfacial regions in the nanocomposite electrode between nanonets of ^*Ag*^NWs and matrix of solution-processable transparent metal-oxide-semiconductor nanomaterials such as zinc oxide nanoparticles (^*ZnO*^NPs) for mechanically strong interface connections as well as efficient heat-dissipating from the nanonets. In addition, ensuring strong adhesions is essential not only within the nanocomposite but also between the nanocomposite electrode and the ultrathin polymer matrix for realizing sturdy architectures of *u*FTEs with smooth surface topography. In the nanocomposite, there are always three different interfaces between (i) ^*Ag*^NWs, (ii) ^*ZnO*^NPs, and (iii) ^*Ag*^NWs and ^*ZnO*^NPs. Although there are various technologies for integrating individual systems of the Ag nanonets at the cross-junctions^27^ and cold sintering of ZnO at the grain boundaries^28^, far more importantly, the heterogeneous contact at the ^*Ag*^NW-^*ZnO*^NP interface requires substantial improvement. One particular concern is the presence of organic residual wrapping on Ag nanonets prevents its direct interaction with ^*ZnO*^NPs^29^. Besides, their non-flexible geometric shapes limit the direct contact area between them^30,31^. As a consequence, there is a primary need to realize the integration of three different interface systems in the nanocomposite electrode of Ag nanonets and matrix of ^*ZnO*^NPs tightly semi-embed in an ultrathin polymer matrix for achieving milestones for the stability of robust *u*FTE structures.

In this study, we demonstrated high operational stability of substrate-integrated *u*FTEs constituted of ^*Ag*^NW nanonets and ^*ZnO*^NP matrix semi-embed in colorless polyimide (cPI) substrates of ultrathin <0.5 μm thickness fabricated via fully solution-processed means. The stable operations against the simultaneous multi-loading fatigue tests were confirmed by retaining a remarkable operational current density of 8.4 MA cm^−2^ flowing through the individual nanowires with only 5 % variation under mechanical folding cycles with 0.5 mm radius of the curvature at 85 % of relative humidity. To achieve it, in-situ solution-processed method (*i*SPM) was developed for both (i) removing the capping agents from ^*Ag*^NWs for direct physical contact of the ^*Ag*^NW-^*Ag*^NW system at the cross-junctions and (ii) facilitating the ^*ZnO*^NPs integration with flexible geometric shapes through the course of the coalescence process of ^*ZnO*^NP-^*ZnO*^NP system. Interestingly, the provided conditions from both (i) and (ii) enable the ^*ZnO*^NPs to be capable of the unprecedented in-situ nano-adhesion into the cleaned surface of ^*Ag*^NWs via the coalescence incidents with flexible geometric shapes in ^*Ag*^NW-^*ZnO*^NP system. The fabricated substrate-integrated *u*FTEs enhanced by the tri-system integration of the above-mentioned ^*Ag*^NW-^*Ag*^NW, ^*ZnO*^NP-^*ZnO*^NP, and ^*Ag*^NW-^*ZnO*^NP systems showed superior electrical/optical/surface roughness properties of sheet resistance of 7.5 Ω sq.^−1^, the average diffused transmittance of >88 % between 400-800 nm of wavelengths when including both electrode and substrate components, and extremely smooth surface roughness with <1 nm of *RMS* and <5 nm of *PtV*. Notably, our results unveiled that the adhesive wetting process of ^*ZnO*^NPs to ^*Ag*^NWs follows the contact line friction model of hydrodynamics with viscous liquid-like behaviour. Consequently, the tri-system integration developed by the *i*SPM offers a general design for smooth topography for the substrate-integrated *u*FTEs achieved via fully solution-processed means with high operational stability of the metal-oxide nanocomposite electrodes.

## Results

### Solution-processed *u*FTEs with four phases of stability

In the metal-oxide composite electrode of ^*Ag*^NWs nanonets and ^*ZnO*^NP matrix, there are always three different types of interface systems including ^*Ag*^NW-^*Ag*^NW, ^*ZnO*^NP-^*ZnO*^NP, and ^*Ag*^NW-^*ZnO*^NP, respectively, hereafter named “the tri-system”. So far, the respective key mechanisms of in-situ integration for both the ^*Ag*^NW-^*Ag*^NW and ^*ZnO*^NP-^*ZnO*^NP interfaces have been intensively studied^32,33^. However, integrating the dissimilar material interface system between ^*Ag*^NWs and ^*ZnO*^NPs is rarely examined in spite of a number of studies on these nanocomposite combinations^21,22^ due to the difficulty to observe their in-situ dynamics for nanoscale interactions between them. Recently, the observation of interacting dynamics between metal nanoparticles and oxide supports in the field of catalysis, which is the opposite geometric concept for this work, has been reported by strong metal-support interaction (SMSI) but the mechanism is not fully understood^34,35^. In particular, the non-adhesive interface between ^*Ag*^NWs and ^*ZnO*^NPs in the field of *u*FTEs will be vulnerable to practical dynamic operating environments such as both repetitive mechanical folding cycles and continuous electrical bias under high humid conditions, making it challenging to harness for modern devices and innovations.

Here, we develop the *i*SPM to achieve the tri-system integration in the metal-oxide composite electrode of ^*Ag*^NWs and ^*ZnO*^NPs for high operational stability against simultaneous multi-loading operations. As for reagents for the *i*SPM, a certain sodium borohydride (NaBH_4_) has been selected for (i) removing capping agents such as polyvinylpyrrolidone (PVP) from the surface of ^*Ag*^NWs, which facilitates direct contact and physical merging at the cross-junctions between the Ag nanonets (^*Ag*^NW-^*Ag*^NW system)^36^ and (ii) allowing flexible geometric shapes of ^*ZnO*^NPs by driving the course of the coalescence process among ^*ZnO*^NPs (^*ZnO*^NP-^*ZnO*^NP system). Interestingly, with the combination of (i) and (ii) after the *i*SPM, the provided conditions between ^*Ag*^NWs and ^*ZnO*^NPs enable the unprecedented adhesive motion of ^*ZnO*^NPs with the coalescence event to the cleansed surface of ^*Ag*^NWs (^*Ag*^NW-^*ZnO*^NP system). The NaBH_4_ concentration of 0.1 molar concentration (M) has been decided for the optimized *i*SPM since it showed a considerable reduction of sheet resistance from the original ones and the rate of the sheet resistance reduction was almost saturated after 0.1 *M* while there was almost no observed silver debris as a by-product (Figure S1).

**Fig. 1:**
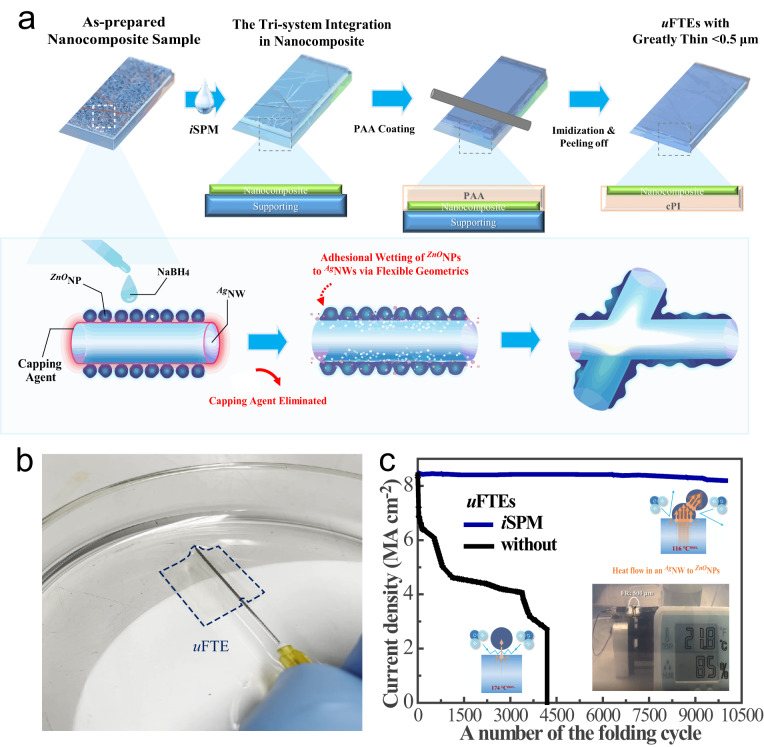
High operation stability of the robust substrate-integrated *u*FTEs with the tri-system integration. **a** A schematic diagram exhibiting the fabrication process of the substrate-integrated *u*FTEs with greatly thin <0.5 μm containing the tri-system integration in the nanocomposite via the *i*SPM. **b** The photo of the *u*FTE (4 cm^2^) floating on the surface of the water getting lifted by a needle. **c**, Real-time monitoring of the operational current density of *u*FTEs according to with (blue) and without (black) the tri-system integration against the simultaneous multi-loading fatigue tests such as the initial 8.4 MA cm^−2^ and 10,000 cycles of repetitive folding (lasting for about 4 hours) with a folding radius of 500 μm under the humid condition of relative humidity 85 %.

**Fig. 2:**
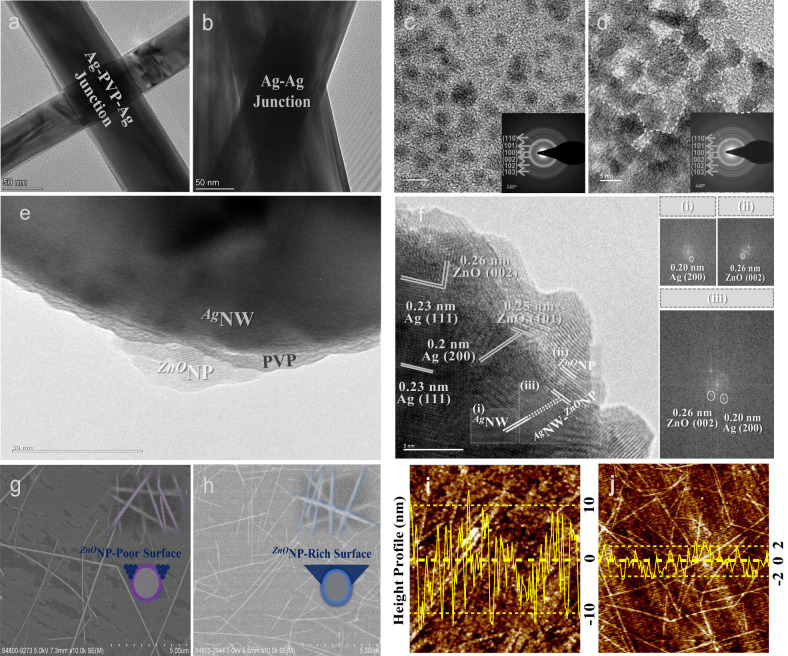
The tri-system integration and extremely smooth surface of the substrate-integrated *u*FTEs via the *i*SPM. The ^*Ag*^NW-^*Ag*^NW interface. **a**, **b** The TEM images illustrating the respective Ag-PVP-Ag and Ag-Ag junctions regarding before and after the *i*SPM, respectively. **c**, **d** The ^*ZnO*^NP-^*ZnO*^NP interface. TEM images of the respective ^*ZnO*^NPs before and after the *i*SPM. The respective insets are the corresponding diffraction patterns. The ^*Ag*^NW-^*ZnO*^NP interface. **e** The as-prepared nanocomposite demonstrating the non-physical connection between ^*Ag*^NWs and ^*ZnO*^NPs in the presence of the PVP layer in the middle. **f** The TEM image magnifying near the interfacial region between ^*Ag*^NWs and ^*ZnO*^NPs. The representative FFT images were extracted from the corresponding (i), (ii), and (iii) boxes, showing an example of the well-matched lattice fringe between ^*Ag*^NWs and ^*ZnO*^NPs. There are many well-matched lattice fringe alignments that can be frequently found. The surface topography of the substrate-integrated *u*FTEs. **g** and **h** The SEM images of *u*FTEs according to with and without the tri-system integration, respectively. The respective insets are the corresponding magnified SEM images, illustrating ^*ZnO*^NP-poor (purple) and -rich (blue) surfaces, respectively. **i** and **j**, The AFM height profiles at the top-surface of the substrate-integrated *u*FTEs according to with and without the tri-system integration, respectively. The respective yellow lines are for measuring the corresponding average height profiles.

Figure 1a depicts the schematic diagram of processing the tri-system integration in the as-prepared metal-oxide nanocomposites on rigid supporting substrates (e.g. silicon wafer, glass, etc.) via the *i*SPM, followed by the entire fabrication progress of semi-embedding the nanocomposite electrode in cPI for the substrate-integrated *u*FTEs with a greatly thin thickness of <0.5 μm. The comprehensive discussions about the respective interfaces can be found in Figure S2 and Fig. 2a–f. The substrate-integrated *u*FTEs with different uniform thicknesses of cPI can be successfully demonstrated by the height regulator of the automatic meniscus printing equipment with an appropriate Mayer bar coating speed (Figure S3). Regarding chemicals for the polyamic acid (PAA) liquid as the precursor of cPI, 2,2′-bis(trifluoromethyl)−4,4′-diaminobiphenyl (TFDB) and 4,4′-(Hexafluoroisopropylidene)diphthalic anhydride (6FDA) have been prepared due to the multi-bulky pendant trifluoromethyl groups. The crucial cPI substrate shows only a low water absorption rate of 0.2 % even after 3 days immersed in water because of the waterproofing effect of the fluorine atoms and to properly screen the thermal stability of *u*FTEs during the multi-loading operations, the substrate requires to have high glass transition temperatures such as the cPI (>300 °C)^37^. Details in the PAA synthesis have been previously reported^20^ and the imidization process of the PAA liquid to the cPI substrate is described in the experimental section. Figure 1b shows an optical photo of the fabricated substrate-integrated *u*FTEs with an extremely thin thickness of <0.5 μm floating on the surface of the water being lifted in a needle. The as-prepared nanocomposite electrodes without the cPI substrate and the cPI substrate-integrated *u*FTE showed an averaged diffused transmittance of >92 and >88 %, respectively. They showed negligible change in optical transparency at wavelengths between 400 nm and 900 nm with and without the *i*SPM (Figure S4), while the averaged reduction of 25 % in electrical sheet resistance to 7.5 ± 1 Ω sq^−1^ for the *u*FTEs was confirmed after the *i*SPM (Table S1). It should be noted the *u*FTEs with the *i*SPM show very good resistance uniformity at different locations of the sample (Figure S5).

We will express the exceptional operations of the robust substrate-integrated *u*FTEs with the tri-system integration in terms of four phases of mechanical-electrical-thermal-moisture stability. Figure 1c exhibits real-time monitoring for the operational current density against the simultaneous multi-loading operations of 10,000 times folding cycles with 500 μm of a folding radius at 85 % relative humidity condition. The current density of 8.4 MA cm^−2^ has been set as the initial current density flowing through the individual nanowires with an averaged diameter of 30 nm^38^, which surpasses the highest reported current densities at about 5 and 5.3 MA cm^−2^ for the previously ^*Ag*^NW-ZnO and ^*Ag*^NW-titanium dioxide (TiO_2_) based electrodes, respectively, so far according to our literature reviews^25,39^. All the tested *u*FTEs were prepared on the supporting cPI with a thickness of 10 μm as a carrier film for the dynamic fatigue tests. From the results, the unstable current density of the optimized *u*FTEs without the tri-system integration was confirmed with a gradual decrease and they eventually exhibited breakdown at the folding cycle of 4-5 K times. In contrast, the robust *u*FTEs with the tri-system integration show remarkable improvement with an insignificant current density variation of less than 5 % under the same operation. Apparently, the improvement could be partially ascribed to the respective ^*ZnO*^NP-^*ZnO*^NP and ^*Ag*^NW-^*Ag*^NW interface integrations of the tri-system. The most difficult part of the technology is  to optimize ^*Ag*^NW-^*ZnO*^NP interfacical interaction in order to complete the tri-system integration. First of all, to clarify the reason for the mechanical degradation regarding the non-adhesive ^*Ag*^NW-^*ZnO*^NP interface, it is proven that many torn surfaces caused by ruptures along the weak interfaces between ^*Ag*^NWs and the adjacent ^*ZnO*^NP matrix were generated at the folded area with a radius of 250 μm (Figure S6b). On the other hand, the robust *u*FTEs with the tri-system integration can preserve the firm surface in the same mechanical folding condition (Figure S6c). On top of that, in conjunction with applying the mechanical folding with a folding radius of 500 μm, the escalated maximum temperatures (*T*) at the most folded edge of the *u*FTEs with different current densities were measured by an infrared thermometer (Figure S6d). Generally, the results show that the detected maximum *T* values from the *u*FTEs with the tri-system integration are always reduced at the same current density as compared to the ones without the tri-system integration. For instance, the *T* of 116 °C at 8.4 MA cm^−2^ was measured from the robust *u*FTEs with the tri-system integration but the ones without the tri-system integration reached the higher *T* of 174 °C at the same current density. The difference between the two measured temperatures implies the critical information that even though Ag nanonets are combined with ^*ZnO*^NPs matrix with the average 5 nm diameters of ^*ZnO*^NPs filling very fine gaps, there is still an inefficient heat-dissipating between ^*Ag*^NWs and ^*ZnO*^NPs at the interface unless direct adhesive binding is involved (Figure S7), which is consistent with the phenomenon of the discontinuous aggregation of titanium oxide nanoparticles to ^*Ag*^NWs with weak attachments^23^. To provide different detailed results into the degradation, the *u*FTEs were tested under the continuous current bias without mechanical stress/strain as shown in Figure S8. Moreover, the high joule heating of Ag nanonets exposed to the high humidity due to the detachment from the ^*ZnO*^NP matrix will further accelerate the degradation of ^*Ag*^NWs by the corrosive reactions^40^. Eventually, our results show that the reason for the broken *u*FTEs without the tri-system integration after the multi-loading fatigue test is caused by the complex mechanical-electrical-thermal-moisture effects on the physically weak ^*Ag*^NW-^*ZnO*^NP interface system, resulting in fatal damages on the most folded area (Figure S9). A trend of sequential quantum drops in operational current density was the direct evidence of the most serious annihilation when the absence of the tri-system integration (Figure S10), meaning that groups of ^*Ag*^NW networks in the nanocomposite electrode were broken down sequentially due to the respective regional hotspot propagations under electrical stress^41^. In addition, we investigated the thermal stress on both *u*FTEs and cPI by characterizing the resistance changes of the *u*FTEs and the SEM images for surface morphology evolution of cPI substrates with elevated temperatures, respectively (Figure S11 and S12). It was found that the *i*SPM-modified *u*FTEs showed less resistance change with temperature particularly when the temperature was higher than 150 °C as compared to the unmodified ones. Yet, there is a concern that after the PVP removals from the ^*Ag*^NW networks, the air exposure could potentially be harmful. To confirm it, the *i*SPM-treated *u*FTEs with of PVP removal were placed in an ambient environment for 35 days. Interestingly, the *u*FTEs with of PVP removal showed stable resistance which could be ascribed to both the passivation by the ^*ZnO*^NPs matrix and fluorinated cPI despite the PVP removals (Figure S13a). Consequently, the <0.5 μm-thick cPI substrate-integrated *u*FTEs have been successfully demonstrated and achieved the sturdy composite electrode of ^*Ag*^NWs and ^*ZnO*^NPs with the *in-situ tri-system integration* for realizing the mechanical-electrical-thermal-moisture (i.e., four phases) stability for the robust and long stability in promising solution-processed *u*FTEs.

### Extremely morphological smooth *u*FTEs with the tri-system integration

We first exhibit the discernible evidence of the tri-system integration before and after the *i*SPM on the respective ^*Ag*^NWs, ^*ZnO*^NPs, and composite of ^*Ag*^NWs and ^*ZnO*^NPs, by investigating the transmission electron microscopy (TEM) images (Fig. 2a–f). Please note that the respective samplings of Fig. 2a–f were made by transferring to the copper grid substrates of TEM from the corresponding samples on glass substrates.

As for the as-prepared ^*Ag*^NWs prior to the *i*SPM, the cross-junction shows the distinct junction of the Ag-PVP-Ag constitution (Fig. 2a). On the other hand, the one after the *i*SPM exhibits the direct Ag-Ag junction at the cross-junction due to the absence of the capping agents (Fig. 2b), which stands for better current flows through the Ag nanonets with the extended electrical pathways. A number of the better conductive cross-junctions can be found with direct contacts and physical mergings between the Ag nanonets (Figure S2d). Regarding the ^*ZnO*^NP morphology after the *i*SPM (Fig. 2d), it is discovered that ^*ZnO*^NPs can lead to physical merges among the ^*ZnO*^NPs, as compared to the ones with the non-interactions between many separate ^*ZnO*^NPs before the *i*SPM (Fig. 2c). The inserted diffraction pattern of Fig. 2d extracted from the corresponding TEM image explicates the particular contribution of the *i*SPM on crystallinities of the merged ^*ZnO*^NPs cannot be found as compared to the ones before the *i*SPM (inset of Fig. 2c)^42^. The impact on the chemical composition was negligible confirmed with no particular chemical composition change before and after the *i*SPM by X-ray photoelectron spectroscopy (XPS) measurements (Figure S14). The observed change in the ^*ZnO*^NPs geometries can also support the higher densification degree of the ^*ZnO*^NP matrix as an increasing number of the *i*SPM (Figure S15). The densely packed ^*ZnO*^NP matrix resulted from the merging ^*ZnO*^NPs after the *i*SPM can provide *u*FTEs with more high heat transfer efficiency due to the enlarged contact areas between ^*ZnO*^NPs (Figure S2e) rather than the loosely packed matrix with air (Figure S2b)^43^.

In the case of the composite of ^*Ag*^NWs and ^*ZnO*^NPs before the *i*SPM, there is the obvious presence of the insulating organic PVP ligand layer sandwiched between ^*Ag*^NWs and ^*ZnO*^NPs, causing the generation of the resistive Ag-PVP-ZnO connections (Fig. 2e). The PVP barrier physically blocked the contact between ^*Ag*^NWs and ^*ZnO*^NPs, leading to weak adhesions along the ^*Ag*^NWs at the interface (Figure S16a). Very differently, there are various atomic arrangement cases of many ^*ZnO*^NPs continuously adherent to the surfaces of an ^*Ag*^NW in the absence of the blocking layer after the *i*SPM (Fig. 2f). Many well-matched lattice fringe alignments can be easily found between (i) Ag (111) and ZnO (002), (ii) Ag (200) and ZnO (101), etc. Additionally, a number of the adhesive interfaces were shown (Figure S16b), verifying that the *i*SPM can create attractive adhesive interactions between the cleaned surface of ^*Ag*^NWs and the flexible geometric shapes of ^*ZnO*^NPs. These evolutions can also contribute to understanding the more closely packed nanocomposite electrode after the *i*SPM (Figure S2f) evolved from the case with many protruding ^*Ag*^NWs before the *i*SPM (Figure S2c). In fact, a large amount of additional ^*ZnO*^NPs could provide the ^*Ag*^NW networks with a well-coverage. However, unless there is a strong attractive adhesion of ^*ZnO*^NPs to ^*Ag*^NWs, the temporary cover by the additional ^*ZnO*^NPs could easily detach from ^*Ag*^NWs whenever applied to dynamic operations. The adhesive interface between ^*Ag*^NWs and ^*ZnO*^NPs can not only explain the mechanically robust nano-adhesion but also the efficient heat-dissipating mechanism from ^*Ag*^NWs to the directly contacted ^*ZnO*^NPs for the use of *u*FTEs. The representative fast Fourier transform (FFT) images are extracted from the corresponding (i), (ii), and (iii) dotted boxes that indicate lattice structures of Ag (200), ZnO (002), and both of them inclusive, respectively (right of Fig. 2f). Based on the referred from the experimental TEM images, an atomistic pattern of the Ag (111) and ZnO (0001) surface is presented to provide the direct support of chemically seamless integrations between these two constitutional materials with the minimal lattice mismatch (Figure S17a)^44^. The XPS results can well explain the replacement of PVP with ^*ZnO*^NP in the direct bonding with Ag (Figure S17b)^45^.

The smooth morphology is well-known as one of the most important requirements in the compatibility with multi-layered electronics. The tri-system integration via the *i*SPM significantly contributed to semi-embedding the smooth surface roughness of the nanocomposite electrode in the cPI matrix (Fig. 2h) as compared to the comparison sample without the tri-system integration (Fig. 2g). The optical microscopic images in dark and phase modes for surface topography of the substrate-integrated *u*FTEs are provided for comprehensive evaluation (Figure S18). The comparison surface exhibited very rough topography with over 4 nm of *RMS* and 20 nm of *PtV* roughness, respectively, (Fig. 2i). The rough surface could be formed between complex situations including the rigid supporting substrate, nanocomposite electrode, and cPI matrix, respectively. For instance, the disconnected ^*ZnO*^NP matrix and weak interaction with Ag nanonets can locally tear apart the nanocomposite electrode. In addition, the PAA liquid precursor can be able to permeate into the loosely packed gaps between ^*Ag*^NWs and ^*ZnO*^NPs within the nanocomposite electrode without the *i*SPM, which hinders the densification of the nanocomposite electrode (Figure S2c). The rough surface can be frequently observed due to the weak binding to both cPI and ^*Ag*^NWs in terms of ripping the ^*ZnO*^NP matrix away from the cPI substrate and protruding ^*Ag*^NWs to the top ^*ZnO*^NP-poor surface (Fig. 2g). A similar phenomenon can consistently be observed in the previously reported work with no tri-system integration^26^. In contrast, the *u*FTEs with the tri-system integration showed an extremely smooth surface topography with <1 nm of *RMS* and <5 nm of *PtV* roughness (Fig. 2j). This can be explained by both the tight-bound ^*Ag*^NW-^*ZnO*^NP composite electrode to the cPI matrix and the strongly adhesive nanocomposite being completely delaminated with ^*ZnO*^NP-rich surface (Fig. 2h). To establish the pragmatic viability of the developed *u*FTEs, the demonstration in real optoelectronic devices such as organic photovoltaics and its comprehensive study are provided in the Supporting Information (Figure S19). As a consequence, the unprecedentedly strong adhesion between ^*Ag*^NWs and ^*ZnO*^NPs has been experimentally demonstrated. The successfully semi-embedded nanocomposite electrode in the cPI matrix showed the extremely smooth surface roughness promoted by the tri-system integration, which played a decisive role in real optoelectronic devices.

### Unveiling the viscous liquid-like behaviour of ^*ZnO*^NPs into ^*Ag*^NWs

Observing the merging dynamics of dissimilar nanomaterials at the heterogeneous interface plays an important role in understanding the formation of complex interface systems^46–48^. In the present section, via the *i*SPM, we discuss the finding of both the in-situ coalescence dynamics among solid-state ^*ZnO*^NPs and the in-situ wetting dynamics into the surface of ^*Ag*^NWs, which will be potentially useful as a general guidance for durable design in various combinations between metal nanowires and transparent metal-oxide-semiconductor nanoparticles. We noticed that the observations of the in-situ dynamics were allowed by the sampling of TEM with natural drying after the *i*SPM directly on the copper grid substrate but without annealing. The *i*SPM could be able to provide ^*ZnO*^NPs with the releasing energy from the deep potential-energy well by substrates in TEM^49^. The coalescence process may be accelerated by the electron beam irradiation in TEM but the limited influence of the irradiation is discussed in Supporting Information.

**Fig. 3:**
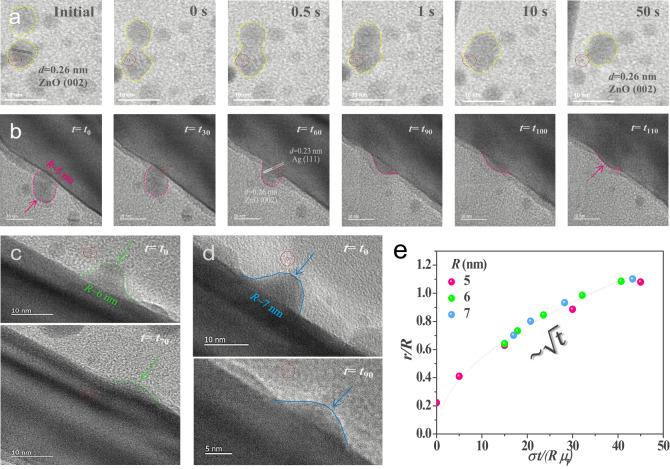
In-situ dynamics of ^*ZnO*^NPs to ^*Ag*^NWs. **a** Sequential TEM images of in-situ dynamics of the representative of two ^*ZnO*^NP pair. After the initial stage that the two ^*ZnO*^NPs are away from each other, and the necking has been set as the standard to start counting at 0 s. The evolving boundary is marked with yellow color. **b** In-situ sequential TEM images of a ^*ZnO*^NP with 5 nm of *R*. The time was started to measure until 110 s from t_0_ (e.g. *t*_110_ = *t*_0_ + 110 s), in which the wetting dynamics were captured from the almost beginning. The *t*_0_ was set on the coalescence event of the ^*ZnO*^NP to the ^*Ag*^NW. The evolving boundary of the ^*ZnO*^NP with *R* of 7 nm is marked with red color. **c**, **d** The respective TEM images of ^*ZnO*^NPs being wetted on the surface of ^*Ag*^NWs measured after 70 s for the estimated 6 nm of *R* and 90 s for the estimated 7 nm of *R* from *t*_0_, respectively, in which t_0_ is the standard to start counting t (e.g. *t*_70_ = *t*_0_ + 70 s and *t*_90_ = *t*_0_ + 90 s). The measurement started to count from the middle of the wetting process of ^*ZnO*^NPs on the surface of ^*Ag*^NWs. The evolving boundary is marked with green and blue for 5 and 6 of *R* in the respective ^*ZnO*^NPs. **e** The plot of *r/R* ~ (*σt/Rμ*_*t*_) interpreting that spreading dynamics of different *R* of ^*ZnO*^NPs for 5 (red), 6 (green), and 7 (blue) nm follows a viscous liquid-like behaviour tha the temporal evolution of t *r/R* is proportional to ~*t*^1/2^.

Figure 3a exhibits the in-situ evolution as the coalescence process between a pair of ^*ZnO*^NPs from the initial stage that the pair is apart from each other (Supplementary Movie 1). The time at 0 s has been set at a necking event between the collided pair after the initial stage. Notedly, the time set for the necking event might not be precise due to the recording frame rate limit. It can be clearly seen that the *i*SPM enables the in-situ coalescence dynamics of the pair of ^*ZnO*^NPs to proceed toward the energetically minimized atomic rearrangement and ended up with a certain round-shaped geometric. They evolved to a bigger size with about 4 nm of diameter than that of the original ^*ZnO*^NP individuals of the initial stage with a diameter of about 3 nm, respectively, in which the evolution preserves the total surface area within error ranges of 5%. Nonetheless, during the evolution, we noted that the crystallinity of one of the ^*ZnO*^NP pair at the initial stage before the collision was (002) and the crystallographic orientation of the merged ^*ZnO*^NP at 50 s also maintained as (002) as well. This phenomenon is similar to the recently reported liquid-like behaviour of metal nanoparticles discussing that the diffusion of atoms on free surfaces quickly reconstructed the original geometry of metal crystal structures^50^, however, our finding here is in relatively more rigid ^*ZnO*^NPs. The in-situ evolution as the coalescence process was not just only observed in a single pair but also in multiple ^*ZnO*^NPs at the same time (Figure S20). Please note that the as-prepared ^*ZnO*^NPs before the *i*SPM retained stably rigid shapes and could not observe the dynamics (Figure S21).

As for the adhesive wetting dynamics between ^*Ag*^NWs and ^*ZnO*^NPs, the existing random coalescence approach of ^*ZnO*^NPs can reach out to the adjacent cleaned surface of ^*Ag*^NWs to initiate the in-situ wetting dynamics for forming the adhesive ^*Ag*^NW-^*ZnO*^NP interface. Figure 3b shows that a ^*ZnO*^NP with a radius (*R*) of 5 nm as a representing case collides with the surface of an ^*Ag*^NW at a certain moment, followed by gradually spreading out on the surface. To offer temporal TEM images, time was started to measure from the arbitrary contact event *t*_0_ between the ^*Ag*^NW and the ^*ZnO*^NP (i.e. *t*_110_ = *t*_0_ + 110 s in the unit of second). Impressively, the ^*ZnO*^NP showed a liquid-like behaviour, in which the spreading process until *t*_110_ can be almost spread out without discrete/abrupt morphological change (Supplementary Movie 2). The same mechanism was found with the other ^*ZnO*^NPs with 6 and 7 nm of the respective estimated *R* and depicted in Fig. 3c and d, respectively, in which time for the respective temporal TEM images were counted from the middle of the in-situ wetting dynamics.

The liquid-like wetting process of the viscous ^*ZnO*^NPs enabled by the *i*SPM on the clean surface of ^*Ag*^NWs is demonstrated by the relation of *r/R* ~ (*σt/Rμ*_*f*_) as illustrated in Fig. 3e, where *r* is the spreading radius, *σ* is the surface energy and *μ*_*f*_ is the friction coefficient^51^. In this analysis, *μ*_*f*_ is chosen as 0.1 as an intrinsic material property of the surface and *σ* varies inversely as the size of ^*ZnO*^NP decreases^52,53^. Coherently, the collapsed lines of Fig. 3e are matched as previously proposed by de Gennes, which is regarded as a viscous liquid-like behaviour that *r/R* should be proportional to ~*t*^1/2^^54^. Notedly, it is different from the ideal liquid as described by Tanner’s law with ~*t*^1/10^^55^. Consequently, the adhesive solid-state ^*ZnO*^NPs acting like a viscous liquid can intensively get wetted into the cleaned surface of ^*Ag*^NWs.

## Discussion

The robust operational stability of the entirely solution-processed substrate-integrated *u*FTEs with greatly thin <0.5 μm has been demonstrated with the *tri-system integration* within the composite electrode of Ag nanonets and ^*ZnO*^NP matrix. The remarkable mechanical-electrical-thermal-moisture stability is featured by small current density fluctuation of less than 5 % against the simultaneous multi-loading fatigue test (10,000 cyclic mechanical folding with folding radius of 0.5 mm, continuous electrical bias of 8.4 MA cm^−2^, and relative humid condition of 85 %). This robustness is attributed to the *i*SPM approach for achieving the unique tri-system integration of (i) ^*Ag*^NW-^*Ag*^NW, (ii) ^*ZnO*^NP-^*ZnO*^NP, and (iii) ^*Ag*^NW-^*ZnO*^NP at the same time. The tri-system integration via the *i*SPM enabled by (i) in-situ removals of organic residuals from ^*Ag*^NWs, (ii) flexible geometric shapes of ^*ZnO*^NPs under the course of in-situ coalescence process between themselves, and (iii) the liquid-like behaviour of solid-state ^*ZnO*^NPs in-situ wetting onto ^*Ag*^NWs. Meanwhile, the nanocomposite electrode was firmly semi-embedded in the plastic matrix to form the substrate-integrated *u*FTEs via fully solution-processed means. As a good *u*FTE candidate harnessing solution-process, the excellent electrical/optical properties when including both electrode and substrate components (7.5 Ω sq.^−1^ of sheet resistance and >88 % of diffused transmittance in the region of wavelengths 400-900 nm) and extremely smooth surface topography (within <1 nm of *RMS* and <5 nm of *PtV* roughness) have been demonstrated. The flexible OSCs on the developed *u*FTEs with and without the tri-system integration were fabricated and the maximum PCE of 13.66 % from the one without the *i*SPM has been substantially improved to that of 16.51 % for ones on the *i*SPM-modified *u*FTEs. Consequently, the strategic design for the lost-cost, solution-processed, and high-throughput *u*FTEs not only offers a comprehensive understanding of the formation mechanisms of the tri-system integration but also advances the integration of hybrid combinations of nano-materials for employing *u*FTEs in real device applications.

## Methods

### Materials and chemicals

Ethanol (AnalaR NORMAPUR(R) ACS, ≥99.8%, VWR Chemicals) was used in this work. ^*Ag*^NWs dispersed in ethanol were bought from ACS Material with a diameter of 30 nm and length of 100-300 μm. ^*ZnO*^NPs used in this work were synthesized according to the previous work^42^. The concentration of 3 mg mL^−1^ of ^*Ag*^NWs and 15 mg mL^−1^ of ^*ZnO*^NPs dispersed in ethanol, respectively, were used for the nanocomposite electrodes. As for the preparation of the *i*SPM, NaBH_4_ powder (ReagentPlus(R), 99%, Sigma-Aldrich) was dissolved for 0.1 *M* of an optimized molar concentration in a mixture of deionized water and ethanol with an 8:2 ratio. The fluorinated cPI used in this work was synthesized according to the previous work^20^. As for the (poly(amic acid) (PAA)) as the viscous precursor for achieving cPI, 2,2’-bis(trifluoromethyl)−4,4’-diaminobiphenyl (TFDB) and 4,4’-(hexafluoroisopropylidene) diphthalic anhydride (6FDA) with multi-bulky pendant trifluoromethyl groups were bought from J&K Scientific LLC. The PAA of the 6FDA:TFDB (1:1) co-polymer structure in a solvent of Dimethylacetamide (DMA) (SuperDry, J&K Scientific LLC). BHT was prepared by mildly stirring for 6 hours.

### Fabrication of the entirely solution-processed *u*FTEs

Step 1: Preparation of the as-prepared ^*Ag*^NWs-^*ZnO*^NPs electrodes.

Rigid supporting substrates of 4 cm^2^ with smooth surface roughness (e.g. silicon wafer, glass, etc.) were cleaned using deionized water, acetone, and ethanol. The cleaned substrates were then treated with ultraviolet-ozone (UVO) for 15 minutes. The ^*Ag*^NWs networks were then formed on the treated substrates using Mayer rod coating or spin-coating. On top of the as-prepared ^*Ag*^NW networks, the ^*ZnO*^NPs was deposited by the same means.

Step 2: The *i*SPM on the as-prepared ^*Ag*^NWs-^*ZnO*^NPs electrodes.

The as-prepared ^*Ag*^NWs-^*ZnO*^NPs samples were treated by dipping them in the *i*SPM for 30 s. After that, the samples were washed with ethanol and deionized water. This *i*SPM process was repeated three times to get the final nanocomposite electrodes with the tri-system integration.

Step 3: Formation of cPI on *i*SPM-treated ^*Ag*^NW-^*ZnO*^NP samples.

On the *i*SPM-treated ^*Ag*^NW-^*ZnO*^NP samples, the PAA precursor solution of the cPI was coated to cover the whole surface by Mayer bar coating. Right after the PAA coating process, the samples will undergo the imidization processing by annealing with a gradual increase of temperature to 200 °C to form cPI. After the imidization processing temperatures, the samples were cooled down to room temperature.

Step 4: Peeling-off process.

After cooling down to room temperature, the substrate-integrated ^*Ag*^NW-^*ZnO*^NP sample in the cPI were submerged in deionized water and then kept for the natural delamination for the formation of the electrode integrated with cPI substrate.

### Fabrication of the flexible organic solar cell devices

Two different pairs of donor and acceptor materials of P3HT:PC_61_BM and D18-Cl:N3 as organic active layers were purchased from Solarmer Materials Inc. 1,2-dichlorobenzene (DCB) anhydrous and chloroform (CF) ACS used for active layer solvents were purchased from Sigma-Aldrich. The solvent 1-butanol for ^*ZnO*^NP solution as an electron transporting layer was bought from J&K Scientific LLC. Polydimethylsiloxane (PDMS) coated on a glass substrate was used as an adhesive film so that the peeled *u*FTEs were stuck to glass/PDMS as rigid supporting adhesive substrates for the flexible OSC device fabrication. All of the OSC devices with two different bulk heterojunction active layers were inverted devices with structures of cathode / ^*ZnO*^NPs as electron transport layer / active layers / evaporated MoO_3_ as hole transport layer / evaporated Ag as anode. The ITO-coated glass substrates of the reference OSCs were cleaned using sequential ultrasonication in deionized water, acetone, and ethanol for 15 minutes, respectively. Glass/ITO was treated by the UVO for 15 minutes. The ^*ZnO*^NPs solution with a concentration of 15 mg mL^−1^ in 1-butanol as electron-transporting layers were deposited on both the *u*FTEs/PDMS/glass and ITO/glass by spin-coating at 3000 rpm for 40 s, followed by drying at 80 °C for 5 minutes. Two different kinds of organic bulk heterojunction active layers with P3HT:PC_61_BM (1:1, 20 mg mL^−1^ in DCB) and D18-Cl:N3 (1:1.2, 12 mg mL^−1^ in CF) were prepared. After transferring samples into the nitrogen-filled glovebox, these two organic active layers were spin-coated on the ^*ZnO*^NPs layers, followed by post-treatment of 1 h slow growth and then annealing at 130 °C for 10 minutes for the P3HT:PC_61_BM film (static dispense at 670 rpm for 40 s, 30 uL), and annealing at 100 °C for 10 minutes for the D18-Cl:N3 film (dynamic dispense at 2500 rpm for 40 s, 17 uL). The devices were then finished by thermal evaporation of 7 nm MoO_3_ and 100 nm Ag under a vacuum of 2 × 10^−6^ Torr in the high-vacuum deposition system with a device area of 0.08 cm^2^.

### Characterization and measurements

The surface topography of the nanocomposites and substrate-integrated *u*FTEs was examined by SEM (Hitachi S-4800), TEM (Philips Tecnai G2 20 S-TWIN and Thermo Scientific Talos F200X G2), Optical microscope (Ti-2E, Nikon), and tapping mode AFM (NT-MDT NTEGRA), respectively. Temperature elevated in the *u*FTEs under electrical bias was measured by an IR thermometer of Fluke. XPS spectra was measured in the ultrahigh vacuum environment using Physical Electronics PHI 5802 with a monochromatic Al Kα X-ray source. The work functions of the ^*ZnO*^NPs films were measured by a SKP5050 scanning KPM (KP Technology Ltd.). The UPS was characterized by using a He discharged lamp (He I 21.22 eV, Kratos Axis Ultra DLD). The sheet resistance of the *u*FTEs was directly measured from a four-point probe approach. The diffused transmission spectra were obtained from a UV/vis. spectrophotometer of Shimadzu. The current density-voltage (J-V) characteristics of all the fabricated OSC devices were measured under a shadow mask with an opening area of 0.05 cm^2^ using Enlitech AM 1.5 G solar simulator with a light intensity of 100 mW cm^−2^ calibrated with a standard silicon reference cell and the data was collected using a Keithley 2635 sourcemeter. As for the Mayer bar equipment, the laboratory-type high-precision printing and coating machine with the built-in Mayer bar (from Peking University Yangtze Delta Institute of Optoelectronics) was used for the ^*Ag*^NW, ^*ZnO*^NP, and PAA coatings. The experimental adjustments have been carried out to both coating speed with 10-50 mm s^−1^ and height distance of less than 100 μm between the bar and the substrates depending on the target thickness and the viscosity of different materials/solutions. Ultraviolet-ozone (UVO) cleaner with a wavelength of 253.7 nm (Jelight Company, Inc.). As for the folding task, we have used home-made folding machines with linear motion guides motorized with stepper motors. Regarding the software, we used the Arduino home-made program to adjust the linear distance with the minimum value of 1 mm. The detailed procedure of the folding task can be divided by 5 steps. Step (i): The *u*FTE samples were loaded to the sample holders with one end of the electrode fixed on the non-moving ground and the other end to the linear movement ground. Step (ii): For the measurement of the electrical properties, the *u*FTE samples were connected using the 3 M electrical contact clips. Step (iii): The initial distance between the two ends of the electrode was set in the program to make the fixed *u*FTE sample straightened out. Step (iv): The moving distance and the number of forward/backward cycles were set in the program for running the folding test. Step (v): The *u*FTE samples were un-loaded after the folding test. A recorded video of the mechanical folding test of the OSC device is provided (Supplementary Movie 3).

### Reporting summary

Further information on research design is available in the Nature Portfolio Reporting Summary linked to this article.

### Supplementary information

Supplementary Information

Peer Review File

Description of Additional Supplementary Files

Supplementary Movie 1

Supplementary Movie 2

Supplementary Movie 3

Reporting Summary

### Source data

Source Data

## Data Availability

All data are available in the main text or the supplementary materials. Source data generated in this study are provided in the Source Data file. Source data are provided with this paper.
