# Supplementary material for: Tri-system integration in metal-oxide nanocomposites via in-situ solution-processed method for ultrathin flexible transparent electrodes
Source: Nat Commun. 2024 Mar 7;15:2070. doi: 10.1038/s41467-024-46243-6 (PMC10920808; doi:10.1038/s41467-024-46243-6)
Supplement: Supplementary file 1 — Supplementary Information [file 41467_2024_46243_MOESM1_ESM.pdf]

Supporting Information for

Tri-system integration in metal-oxide nanocomposites via in-situ solution processed method for ultrathin flexible transparent electrodes

John Jinwook Kim[1], Kojima Shuji[1], Jiawei Zheng[1], Xinjun He[1], Ahmad Sajjad[1], Hong Zhang[2], Haibin Su[3], Wallace C.H. Choy[1]

1. Department of Electrical and Electronic Engineering, The University of Hong Kong, Pokfulam Road, Hong Kong, China

2. State Key Laboratory of Photovoltaic Science and Technology, Shanghai Frontiers Science Research Base of Intelligent Optoelectronics and Perception, Institute of Optoelectronics, Fudan University, Shanghai, 200433, China

3. Department of Chemistry, The Hong Kong University of Science and Technology, Clear Water Bay, Kowloon, Hong Kong, China

E-mail: chchoy@eee.hku.hk (W.C.H.C.), haibinsu@ust.hk (H.S.)

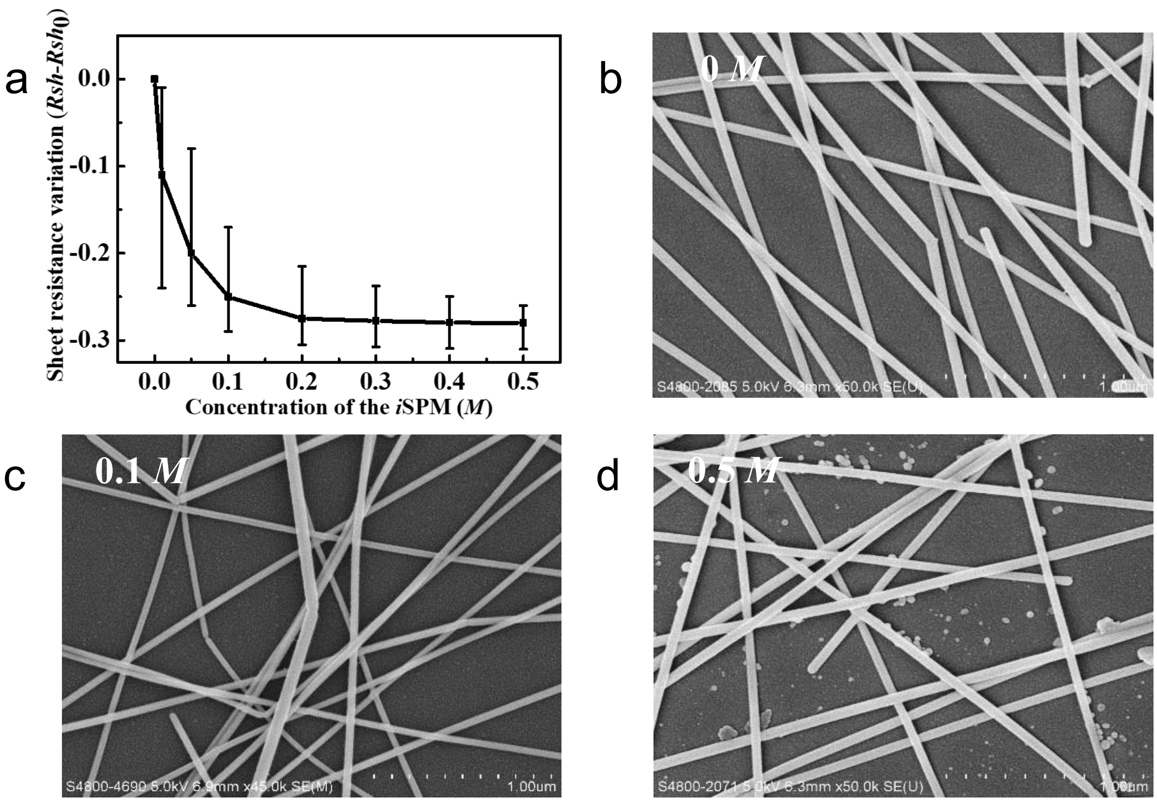

Figure S1 Ag nanonets before and after the *i*SPM with different molar concentrations (*M*) of $NaBH_4$. In the process of the *i*SPM, the Ag nanonets placed on the supporting substrates had been dipped in the prepared $NaBH_4$ solution for 30 s. a, The averaged reduction of sheet resistance (*Rsh*) with the respective error bars over 20 samples of Ag nanonets with the *i*SPM at different concentrations of $NaBH_4$ from 0 to 0.5 *M*. The rate of the reduction is almost saturated when $NaBH_4$ concentration is higher than 0.1 *M*. **b-d** SEM images of the Ag nanonets with the *i*SPM at different concentrations of 0, 0.1, and 0.5 *M*. **b**, 0 *M*. **c**, 0.1 *M*. **d**, 0.5 *M*. It was observed that there were many Ag nanoparticles produced by the highest concentration of 0.5 *M* likely due to the furious reduction reaction with Ag ions on the surface of ^{Ag}NWs. The optimized concentration for the *i*SPM was decided as 0.1 *M* with the consideration of the Ag debris production that there was no Ag debris together with decent sheet resistance reduction when it was 0.1 *M*.

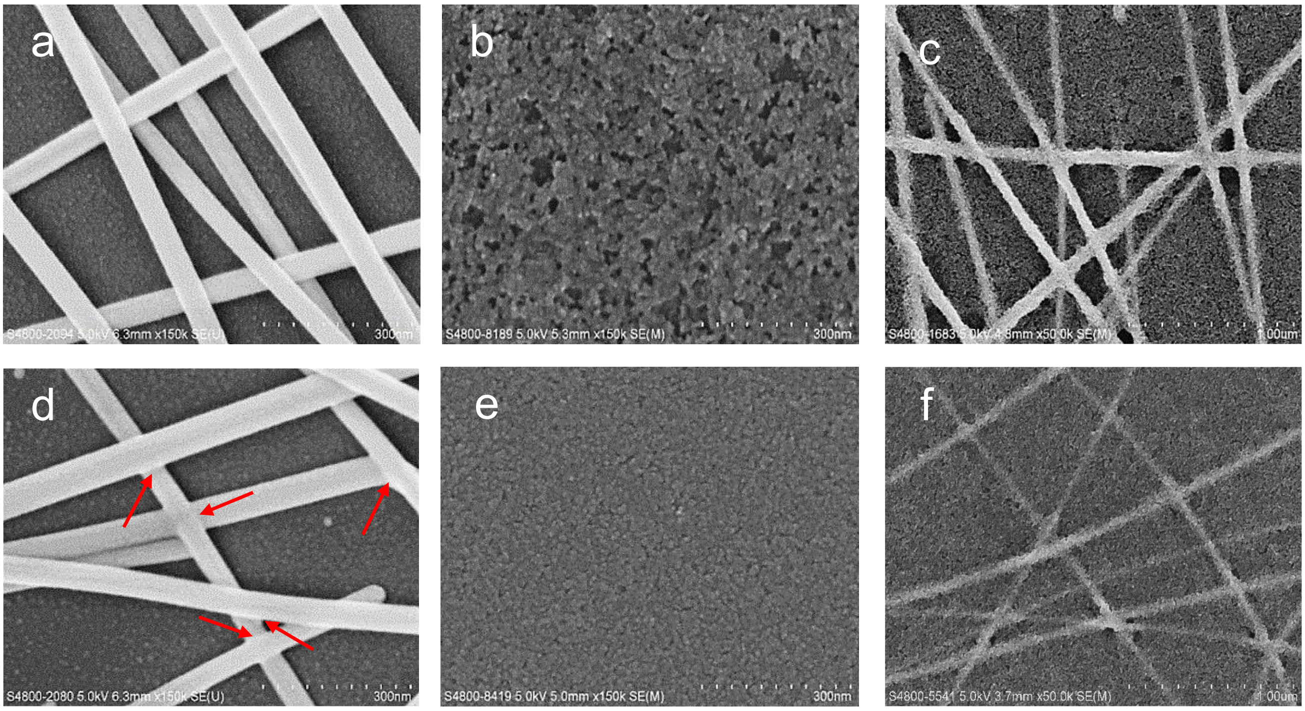

Figure S2 The tri-system integration and morphological evolution of the composite of Ag nanonets and ^{ZnO}NP matrix via the *i*SPM. a-f, SEM images of Ag nanonets, ^{ZnO}NP matrix, and the composite electrode of Ag nanonets and ^{ZnO}NP matrix, respectively, regarding before and after the *i*SPM. **a** and **d**, The before and after of Ag nanonets. The red arrows are pointed for physical merges at the cross-junctions. **b** and **e,** The before and after of ^{ZnO}NP matrix. **c** and **f,** The before and after of the nanocomposite electrode.

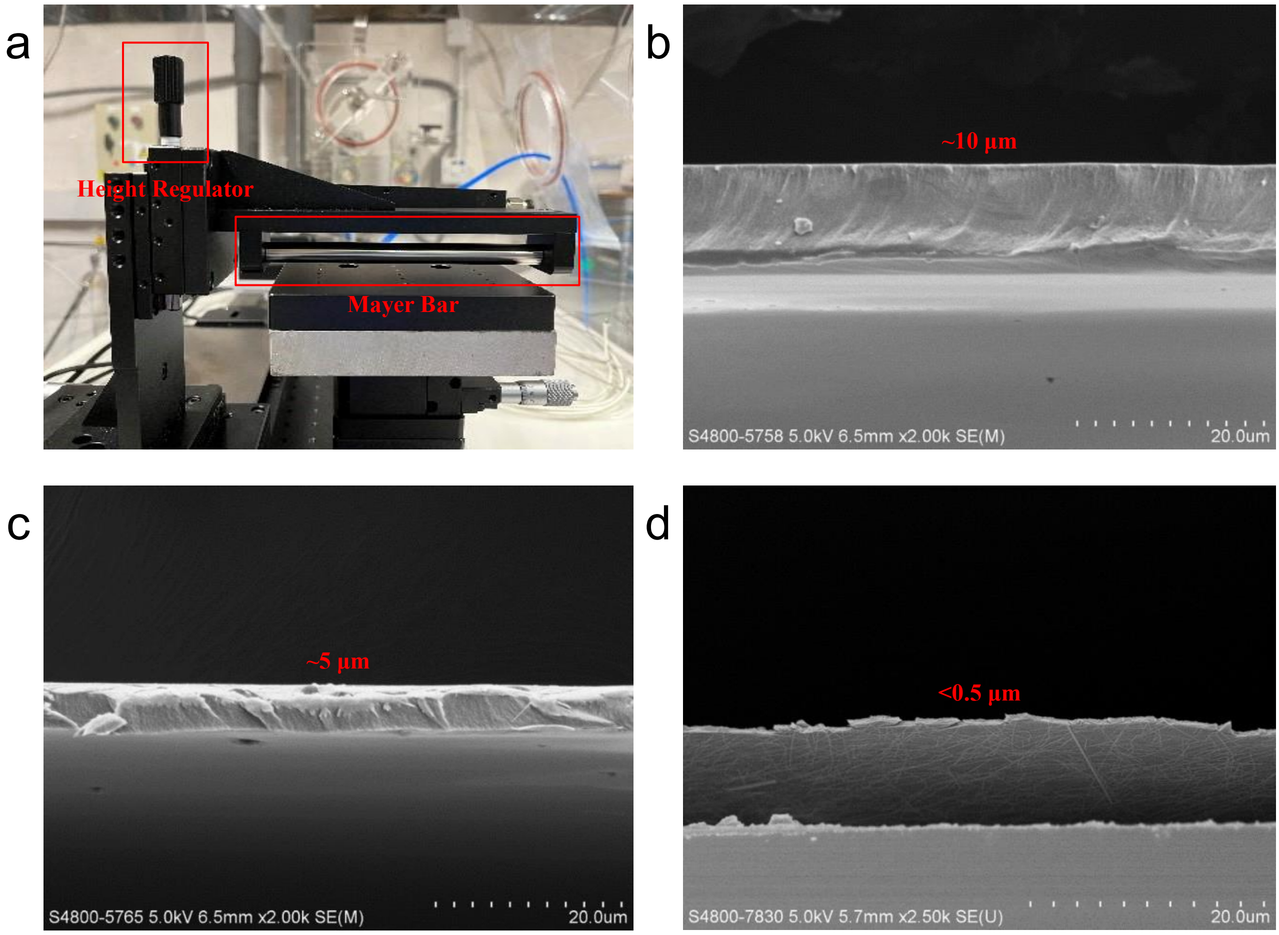

Figure S3 Finely adjustable cPI substrate with various uniform thicknesses. a, The photo of the automatic meniscus printing equipment with a Mayer bar and height regulator. The cPI thickness can be accurately adjusted with the equipment. **b-d**, The cross-sectional SEM images showing the different thicknesses of the substrate-integrated *u*FTEs. **b**, ~10 μm. **c**, ~5 μm. and **d**, <0.5 μm.

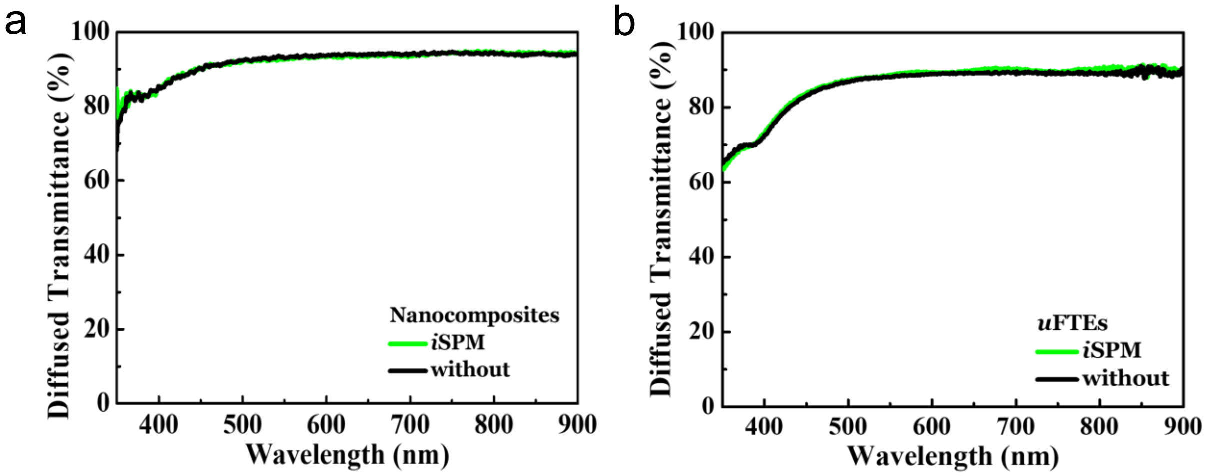

Figure S4 The optical transparency of the as-prepared nanocomposite electrode without cPI substrate and the substrate-integrated *u*FTEs including both the electrode and substrate components. The diffused transmittance spectra regarding with (green) and without (black) the *i*SPM, respectively. **a**, The nanocomposite electrode excluding the cPI substrate. **b**, The substrate-integrated *u*FTEs including both the electrode and substrate components. Almost negligible change in optical properties before and after the *i*SPM is confirmed.

Table S1 A summary of the average sheet resistance and diffused transmittance (stable over the wavelengths between 400 and 900 nm) over 20 samples of the composite electrode of Ag nanonets and ^{ZnO}NP matrix excluding the cPI substrate and the cPI substrate-integrated *u*FTEs before and after the *i*SPM, respectively.

***i*SPM**	**Before**		**After**	
**Sample**	**Sheet resistance**	**Diffused transmittance**	**Sheet resistance**	**Diffused transmittance**
Nanocomposite	10 ± **1 Ω sq^{-1}**	92.9 %	7.5 ± 1 **Ω sq^{-1}**	92.7 %
*u*FTEs	10 ± **1 Ω sq^{-1}**	88.6 %	7.5 ± 1 **Ω sq^{-1}**	88.3 %

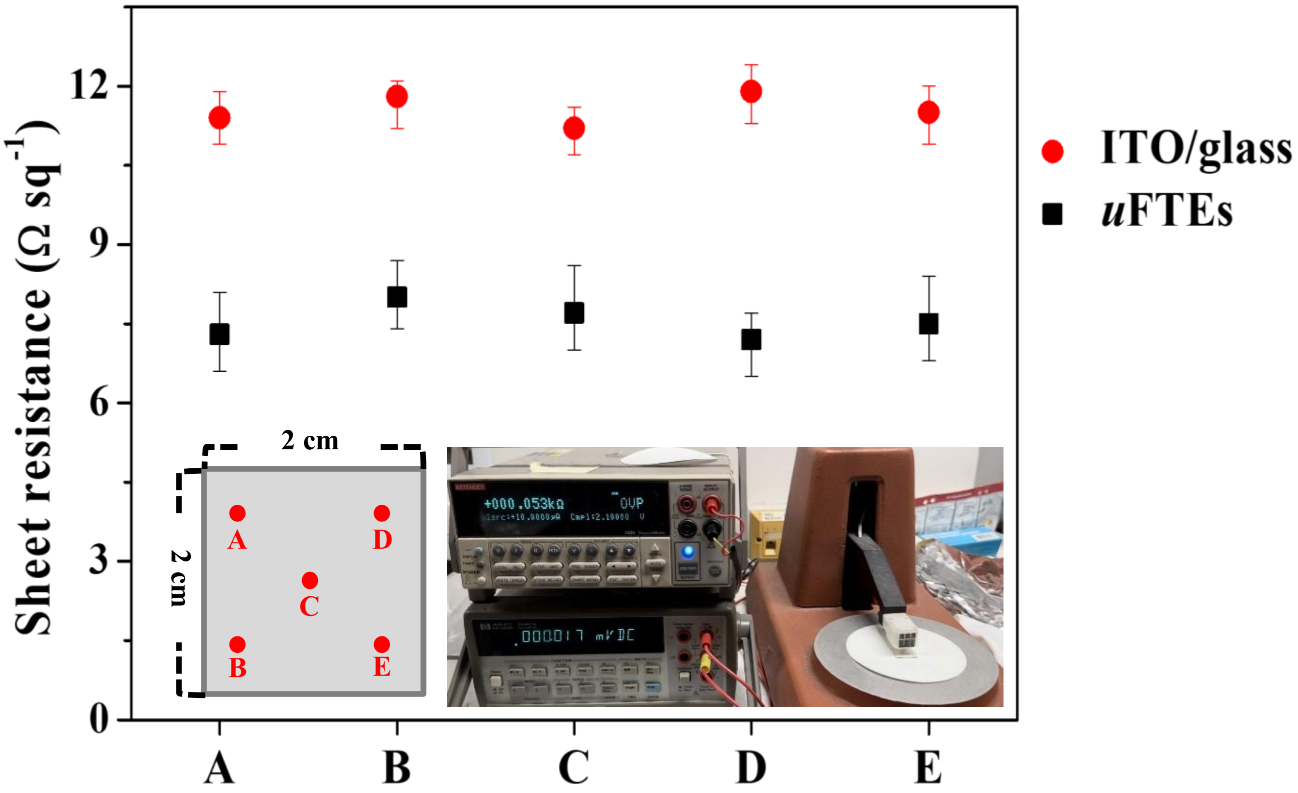

Figure S5 The sheet resistance uniformity of the *u*FTEs. The sheet resistance uniformity at different positions of the *i*SPM treated *u*FTEs measured by a four-point probe (black square). The ITO/glass is added as a comparison (red circle). The respective error bars are inserted by the repetitive measurements with 20 times. The thickness of commercial ITO film on a glass substrate and the conductive ^{Ag}NW-^{ZnO}NP film in *u*FTE is about 150 and 60 nm, respectively.

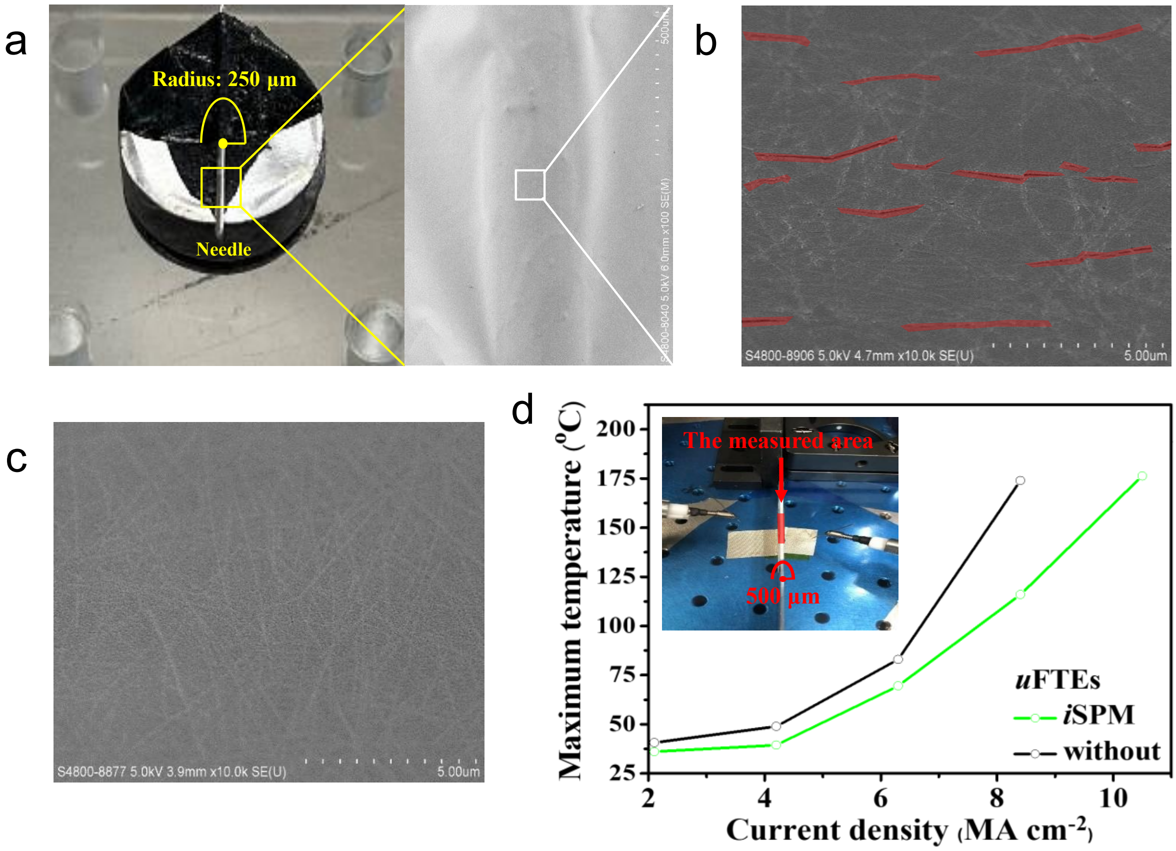

Figure S6 The mechanical and electrical stability of the substrate-integrated *u*FTEs. a, The right SEM image of the wrapping of the substrate-integrated *u*FTE on 250 μm of a folding radius of the needle. The left photo represents the real SEM experimental setting. **b**, The SEM image of the mechanically weak *u*FTE without the tri-system integration at the folded area of the corresponding white box from the SEM of (a), generating the surface cracks (red) of the folded *u*FTE. **c**, The magnified SEM image of the robust *u*FTE with the tri-system integration on 250 μm of a folding radius of the same needle, holding the firm surface at the folded area. **d**, The respective measured maximum temperatures from the *u*FTEs under various current biases from 2.1 to 10.5 MA cm^{-2} at the most folded line by a folding radius of 500 μm according to with (green) and without (black) the tri-system integration. The *u*FTEs without the tri-system integration could not sustain the high current density of 10.5 MA cm^{-2}, resulting in the failure of the temperature measurement. The inset photo represents the measurement set-up for the most folded line of *u*FTEs. The most folded line is highlighted by the red area. The *u*FTEs were connected by a conductive tape for the electrical contact.

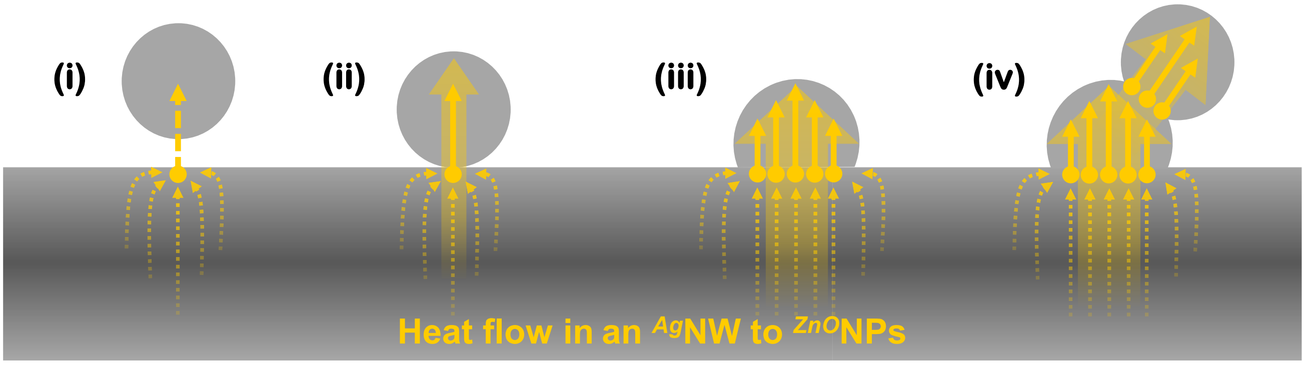

Figure S7 The schematic of four different types of the possible heat flow pathway from ^{Ag}NWs to adjacent ^{ZnO}NPs, respectively. The efficiency order for the respective heat flows will be (i) < (ii) < (iii) < (iv), which has been drawn based on the extended contact area in terms of the directly bridged heat flow pathway [1]. For example, cases (i) and (ii) will typically belong to the one without the interface integration under the respective situations; (i) when mechanical folding is applied and (ii) weak adhesive interactions between ^{Ag}NWs and ^{ZnO}NPs due to impurity barriers or geometrical limits. As for the substantial reduction of joule heating involved in the *u*FTEs with the interface integration, the (iii) and (iv) can be adapted to strong adhesion not only between ^{Ag}NWs and ^{ZnO}NPs for (iii) but also the additional adhesion between ^{ZnO}NPs themselves for (iv). The realization of cases (iii) and (iv) in the nanocomposite electrode can be originated from both the clean surface of ^{Ag}NWs without impurities and the highly adhesive ^{ZnO}NP groups with flexible geometric shapes both to the clean ^{Ag}NWs and among themselves. Accordingly, the efficient heat-dissipating in the *u*FTEs can be explained.

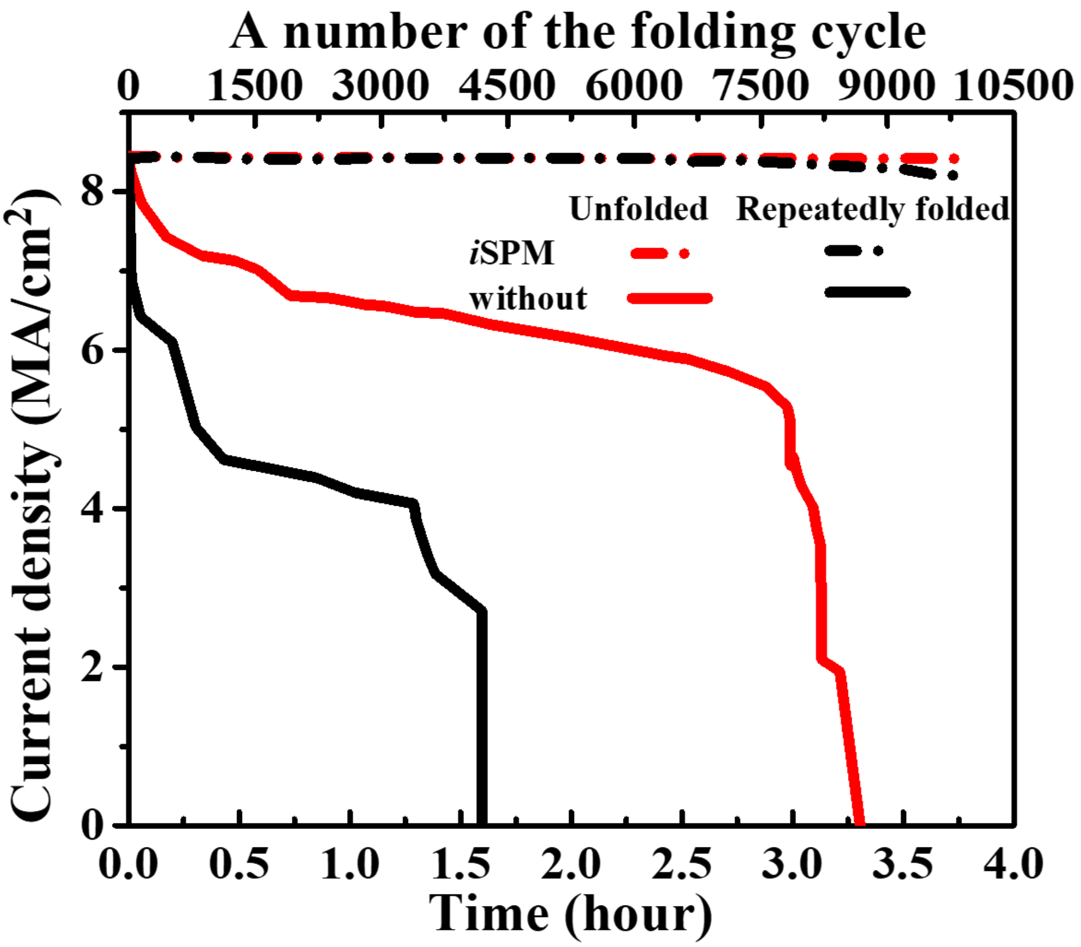

Figure S8 Real-time monitoring of the operational current density of the *u*FTEs with and without the *i*SPM under continuous current bias without mechanical folding. The measurement of the operational current density of the *u*FTEs with (dotted line) and without (solid line) the *i*SPM under no mechanical stress/strain (unfolded by red). The *u*FTEs were flattened by glass-supporting substrates and biased with the initial current density of 8.4 MA cm^{-2} for about 4 hours. For comparison, the results of Figure 1c were plotted together (repeatedly folded by black). Overall, between the samples without the *i*SPM, the repeatedly folded sample showed a relatively much shorter lifetime until they broke down as compared to the unfolded sample. On the other hand, the poor stability of the repeatedly folded sample has been significantly improved after the *i*SPM and showed similar results to the unfolded sample with the *i*SPM.

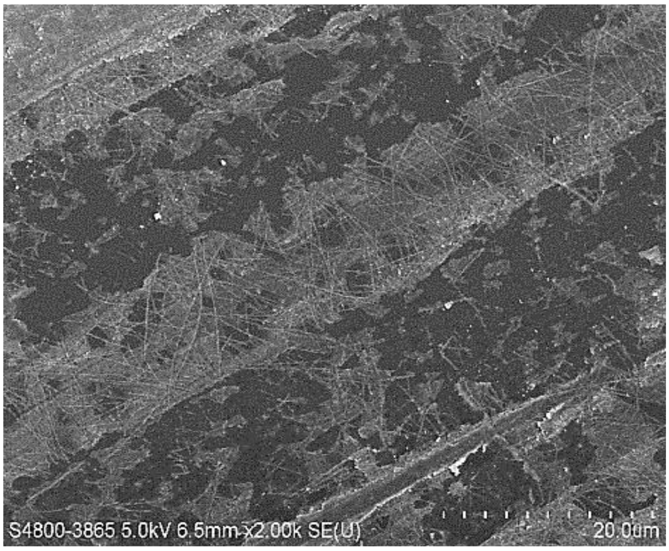

Figure S9 The damaged region of the substrate-integrated *u*FTE without the tri-system integration after the simultaneous heavy multi-loading fatigue tests. The SEM image of the decomposed edge area for the substrate-integrated *u*FTE after the simultaneous heavy multi-loading fatigue tests such as the constant current bias of the initial 8.4 MA cm^{-2} and the simultaneous 10,000 times of the folding cycle with 0.5 mm of a folding radius under 85 % of relative humidity condition. The damaged region due to the complex and heavy loadings commonly shows the completely annihilated nanocomposite electrode along the most folded area.

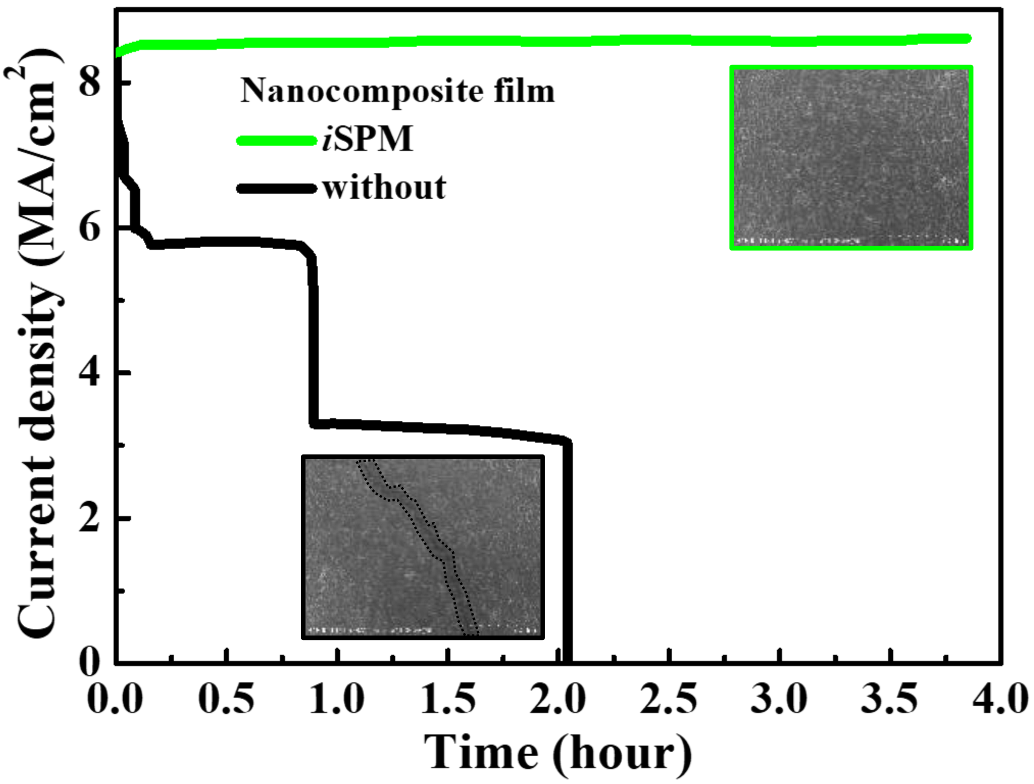

Figure S10 Real-time monitoring of the operational current density of the nanocomposite electrode under continuous current bias regarding with and without the tri-system integration. The measurement of the operational current density from the nanocomposite film. The composite electrodes were coated on glass substrates and biased with 8.4 MA cm^{-2}. The nanocomposite electrode without the tri-system integration (black) could not hold the electrical flow through the individual groups of Ag nanonets in specific regions for a long period, accordingly, exhibiting the sequential quantum drops of the operational current density. The SEM image of the inset in a black box showed the hotspot propagation highlighted by a black color, caused by the joule heating effect during the current flow. In contrast, the nanocomposite electrode with the tri-system integration (green) was found to be remarkably stable. We could not find any degradation and hotspot propagation regions over the whole surface shown in the SEM image of the inset in a green box due to the high thermal management with efficient heat-dissipating of the adhesive interface between Ag nanonets and the ^{ZnO}NP matrix.

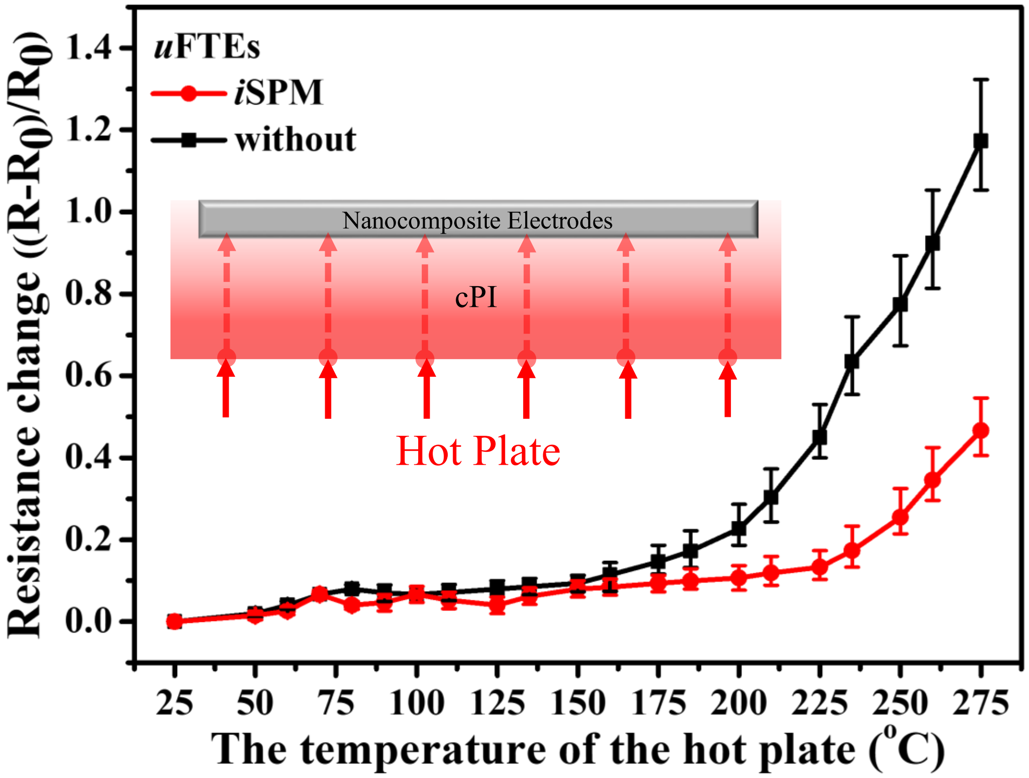

Figure S11 The ratio of resistance changes of the *u*FTEs with and without the *i*SPM against the temperature applied to the cPI substrate. The resistance evolution with (red) and without (black) the *i*SPM was directly measured from the *u*FTEs when the samples were put a hot plate at elevated temperatures for 5 minutes. The respective error bars are presented by the repetitive measurements with 20 times.

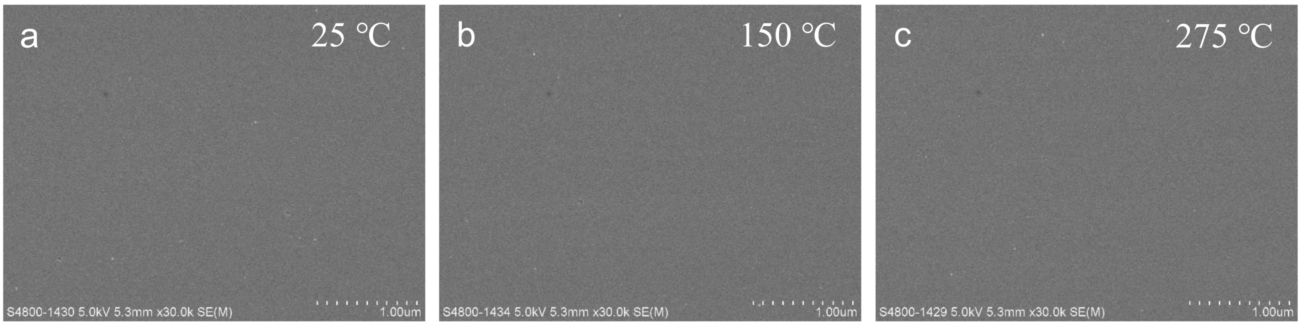

Figure S12 The surface morphology evolution of cPI after exposure to different temperatures on a hot plate. The SEM images after exposure to **a**, room temperature of 25 ℃. **b**, 150 ℃. **c**, 275 ℃. No trace of damage, distortion, or deformation are observed after the thermal test with temperatures as high as 275 ℃. The cPI flexible substrates were tested by directly placing on a hot plate at the designed temperatures for 30 minutes.

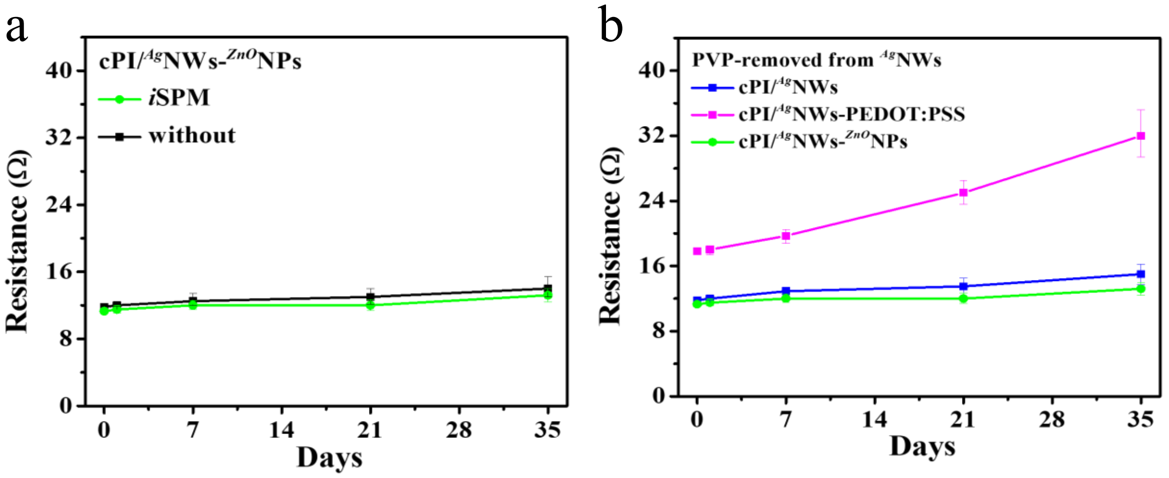

Figure S13 The resistance variation of cPI semi-embedded *u*FTEs under 35 days in air. a, The evolution of the resistance in cPI semi-embedded ^{Ag}NWs-^{ZnO}NPs electrodes according to with (green) and without (black) the *i*SPM. Generally, both the as-prepared ^{Ag}NWs passivated by the PVP ligands before the *i*SPM and the ^{Ag}NWs protected by the ^{ZnO}NP matrix after the *i*SPM showed stable resistance retention for 35 days of air exposure. It should be noted that the exposed areas of both the *u*FTEs to air are very narrow due to the semi-integration by the fluorinated cPI. **b**, The resistance of both the previously reported ^{Ag}NWs (blue) and ^{Ag}NW-PEDOT:PSS (pink) electrodes semi-embedded in cPI after the PVP-removal from the ^{Ag}NW surface was measured and compared to the PVP-removed ^{Ag}NW-^{ZnO}NP electrode of this work (green). The PVP-removals for the bare ^{Ag}NWs and ^{Ag}NWs-PEDOT:PSS were prepared according to reference [2] and [3] respectively. Overall, the PVP-removed ^{Ag}NWs showed stable resistance retention properties, which could be ascribed to the protection of semi-embedding in cPI while the ^{Ag}NW-^{ZnO}NP of this work showed a better protection through the coverage of ^{ZnO}NP matrix to ^{Ag}NWs. However, the retention resistance properties of the composite electrode of ^{Ag}NWs-PEDOT:PSS exhibited gradual degradation during the test period of 35 days due to the acidic properties of PEDOT:PSS. The respective error bars in both a and b are inserted based on the repetitive measurements with 20 times.

.

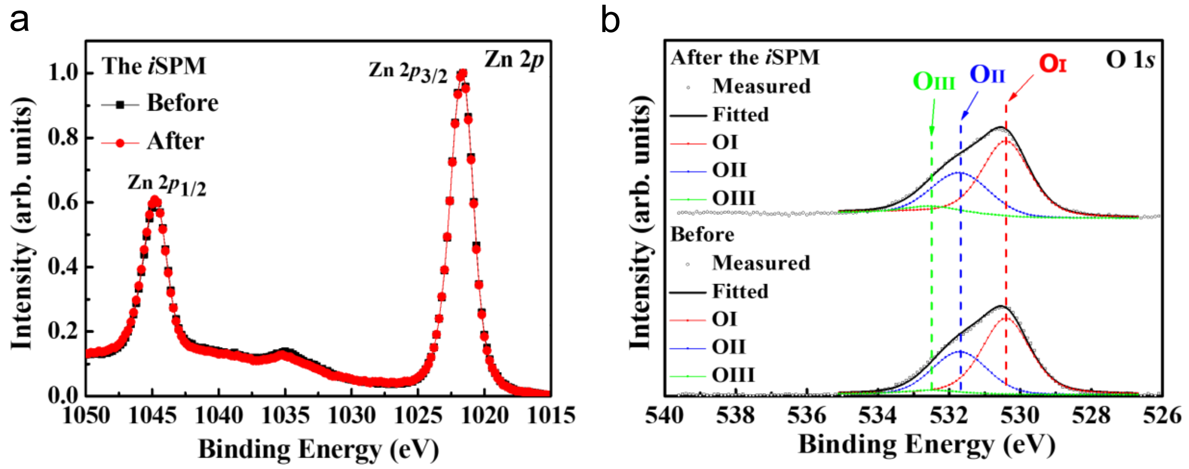

Figure S14 Chemical composition in the ^{ZnO}NP matrix. a and **b**, The XPS spectra of Zn 2*p* and O 1*s*, respectively, for the ^{ZnO}NP matrix regarding before and after the *i*SPM. It can be agreed that there is no chemical composition change after the *i*SPM but a significant morphological change was found.

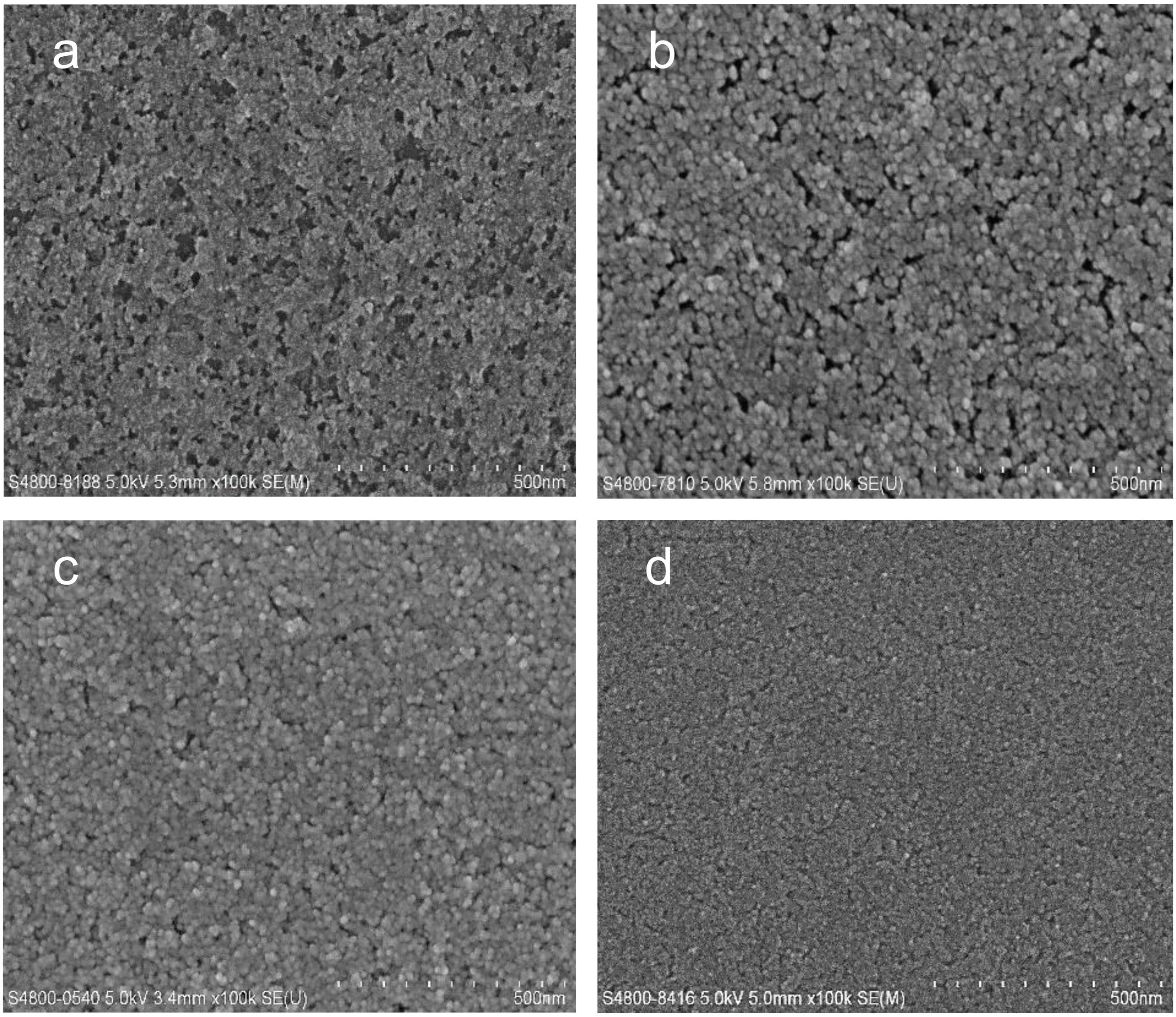

Figure S15 ^{ZnO}NP matrix before and after the *i*SPM with a number of times. a-d, SEM images of the ^{ZnO}NP matrix regarding after the *i*SPM with 0.1 *M* of $NaBH_4$ and a number of times. **a**, before. **b**, after 1st time. **c**, 2nd time. **d**, 3rd time. The densification of the ^{ZnO}NP matrix can be observed and become more compact with an increasing number of the *i*SPM times.

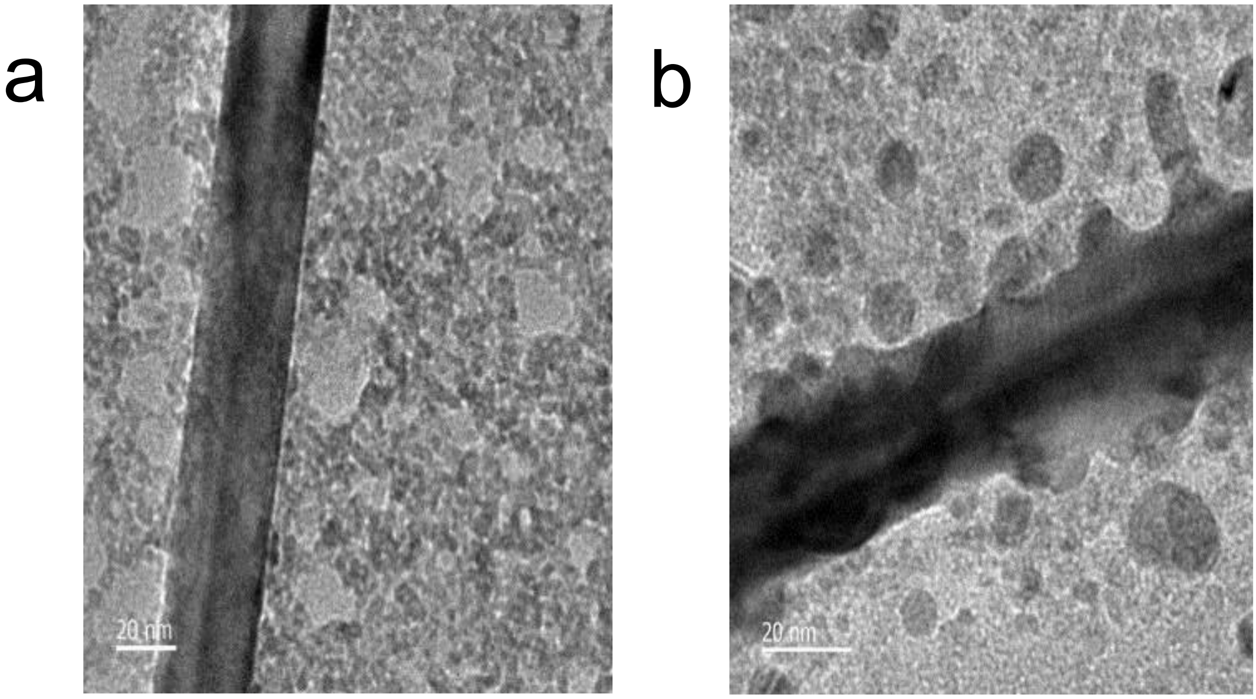

Figure S16 The adhesive interface integration between ^{Ag}NWs and ^{ZnO}NPs via the *i*SPM. a and **b**, The nanocomposite exhibiting the different interfaces between ^{Ag}NWs and ^{ZnO}NPs in terms of adhesive integration regarding before and after the *i*SPM, respectively. The sampling of (b) has been done by consecutive dispersions of ^{Ag}NWs, ^{ZnO}NPs, and the *i*SPM directly on the TEM copper grid substrate while the same sampling for (a) was carried out except for the *i*SPM.

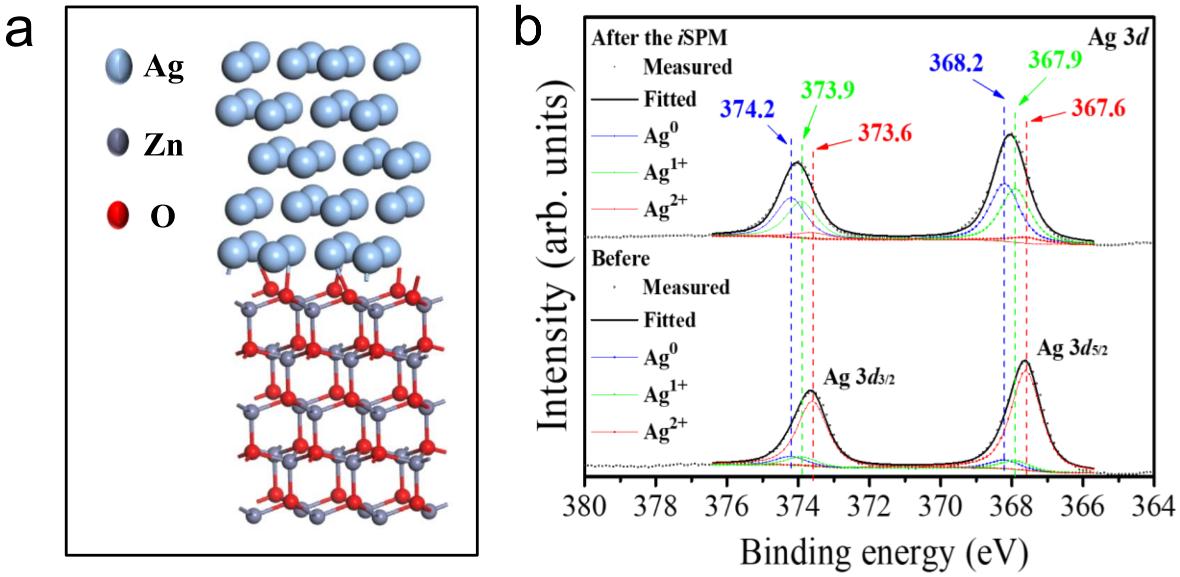

Figure S17 The chemical bonding model and chemical composition in the composite film of Ag nanonets and ^{ZnO}**NP matrix. a**, A chemical structure model of the Ag(111)/ZnO(0001) surface. The surface of O-terminated ^{ZnO}NPs and the surface of ^{Ag}NWs can be more stabilized due to the direct bonding between them. **b**, XPS spectra of Ag 3*d* for the nanocomposite film before and after the *i*SPM. The blue shift of Ag 3*d* peaks after the *i*SPM strongly imply that the Ag^0 (blue) and Ag^{1+} (green) peaks appeared as compared to the case without the *i*SPM due to the reduction of Ag ions and the replacement of PVP by ^{ZnO}NPs, respectively. Meanwhile, the Ag^{2+} (red) peak disappeared due to removal of the PVP ligand from ^{Ag}NWs via the *i*SPM.

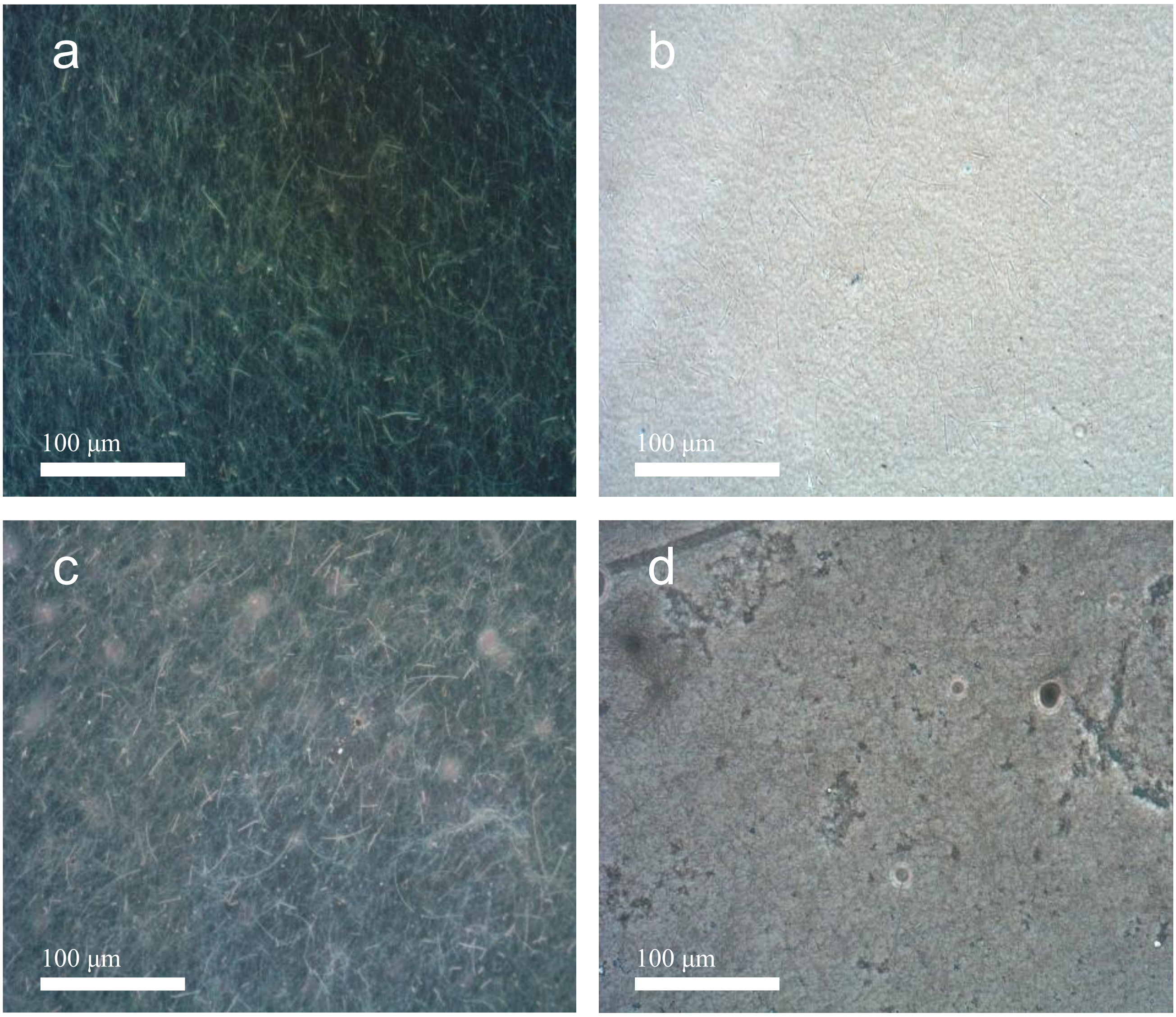

Figure S18 Optical microscopic images for surface topography of the substrate-integrated *u*FTEs. a and **c**, The dark mode for the *u*FTEs with and without the *i*SPM, respectively. **b** and **d**, The phase mode of the corresponding *u*FTEs. The poor coverage of ^{ZnO}NPs of the *u*FTEs without the *i*SPM can be seen (Figure S18d), which is similar results to the SEM image (Figure 2g) of the same *u*FTEs while the tri-system integration plays a decisive role in reducing the surface roughness of the *u*FTEs with the *i*SPM (Figure S18b), which is quite consistent with the SEM image (Figure 2h).

Performance and characterization of *u*FTEs in real optoelectronic devices

To establish the pragmatic viability of the *u*FTEs, flexible organic solar cells (OSCs) fabricated on the *u*FTEs and their comprehensive study are presented in Figure S19. The *u*FTEs with a thickness of 10 μm were utilized due to the vulnerability of the extremely thin cPI (e.g. <0.5 μm) to chemical stability (e.g. dichlorobenzene, chloroform, etc.) and to ease the fabrication of OSCs. Figure S19a demonstrates that for OSCs with D18-Cl:N3 as the active layer the tri-system integration of the *u*FTEs improve the power conversion efficiency (PCE) from 13.66% (without the *i*SPM) to 16.51% which is comparable to that of 16.70 % based on rigid ITO/glass. The short-circuited current (J_{SC}), open-circuit voltage (V_{OC}), fill-factor (FF), and PCE are listed in Table S2. The improvement of the *i*SPM samples could be explained by three reasons. Firstly, it can be accessed by the suppressed leakage current (reverse bias regions of Figure S19b). Secondly, the series resistance reduces (forward bias regions after 0.5 V in Figure S19b). Lastly, it could be described by the modified surface work function value from 3.94 (without the *i*SPM) to 4.14 eV (with the *i*SPM) as calculated from the measured ultraviolet photoelectron spectroscopy (UPS) cut-off energy (Figure S19c). The small energy offset can be expected between the modified *u*FTEs and 4.20 eV of the work function of the bare ^{ZnO}NP film used as the electron transport layer of the OSC devices measured by kelvin probe microscope (KPM) (Table S3). As compared to the other reported ^{Ag}NW-based electrodes with different work functions (Table S4), the *u*FTEs with the tri-system integration could favor interface carrier transportation with a small energy offset for photovoltaic applications [4]. Notably, the PCE of the OSCs on the *u*FTEs with the *i*SPM has been preserved within 10 % after 10,000 times of folding cycles with a folding radius of 1 mm under the continuous AM 1.5G illumination (Figure S19d). The decreased PCE was closely related to the reduction of J_{SC} and FF while V_{OC} remained similar (Figure S19e). The video of the mechanical folding test of the OSC device conducted in the glovebox filled with nitrogen gas is provided (Supplementary Movie 3).

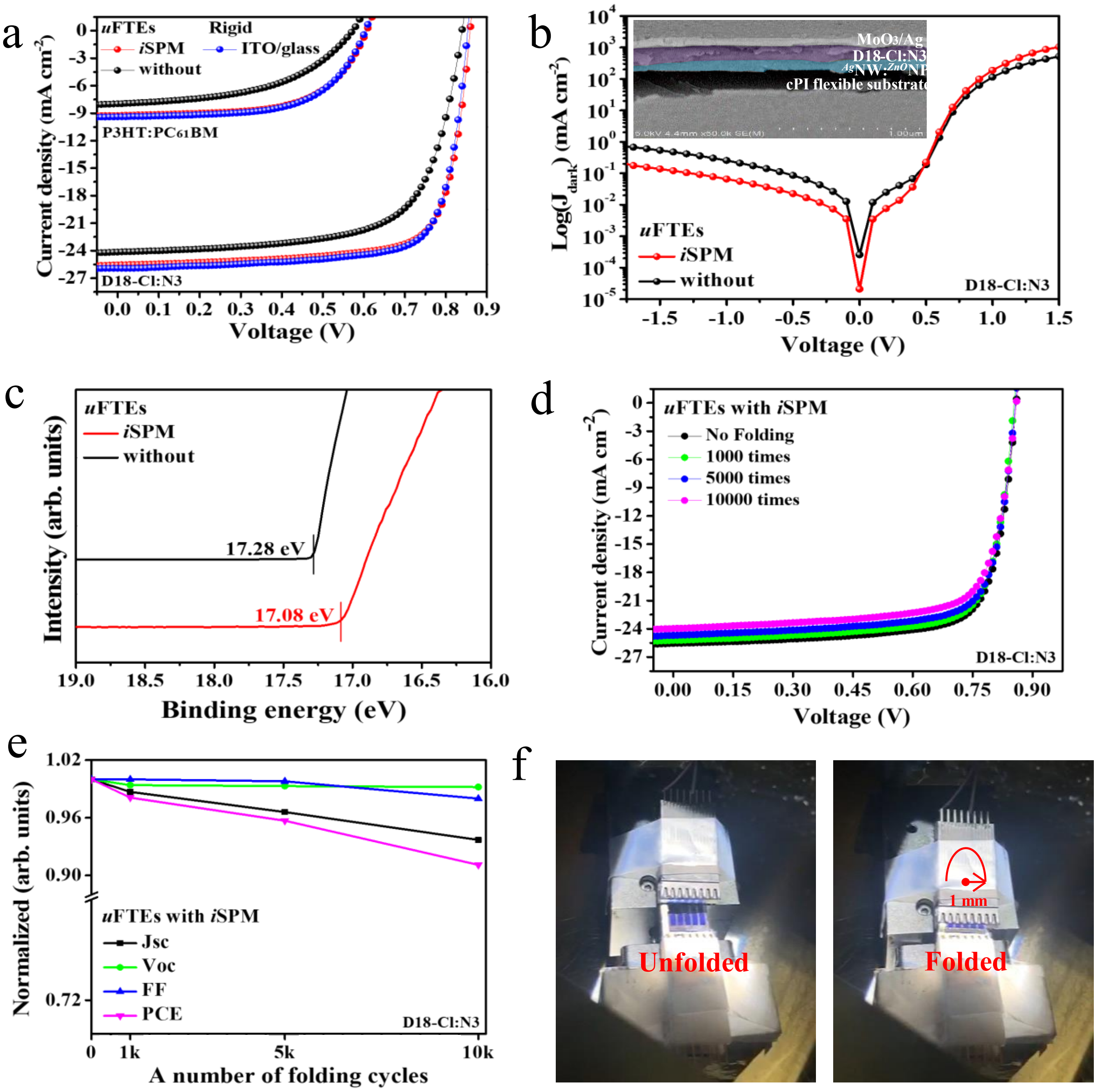

Figure S19 Performances of the flexible OSCs on *u*FTEs with and without the *i*SPM. a, The J-V curves of the optimized flexible OSCs with P3HT:PC_{61}BM and D18-Cl:N3 as active layers under the AM 1.5G illumination according to with (red) and without (black) the *i*SPM. The J-V curves of the reference OSCs on rigid ITO/glasses is also provided (blue). **b**, The J-V curves under the dark state of the flexible OSCs with the D18-Cl:N3 active layer with (red) and without (black) the *i*SPM. The leakage current has been suppressed by the *i*SPM. In addition, the series resistance of the *i*SPM case is improved as displayed by the higher current density at a forward electrical bias over 0.5 V. The inset shows the SEM image of a cross-section of the optimized flexible OSC with the *i*SPM. **c**, UPS spectra of the *u*FTEs in the cut-off regions. The calculated work function value increases to 4.14 (with the *i*SPM, red) from 3.94 eV (without the *i*SPM, black). **d**, The evolved J-V curves of the flexible OSC based on the *i*SPM-treated *u*FTE at 1,000 (green), 5,000 (blue), and 10,000-time (pink) mechanical folding cycles under the continuous AM 1.5G illumination. The folding radius is 1 mm. **e**, The

evolution of the normalized J_{SC} (black), V_{OC} (green), FF (blue), and PCE (pink) measured from the J-V curves of Figure S19d for the respective mechanical folding cycles. **f**, Optical images of the flexible OSCs at the unfolded (left) and folded (right) states in the mechanical folding test with a folding radius of 1 mm under the AM 1.5G illumination. All the measurements were carried out in the glove box filled with a nitrogen environment.

Table S2. A summary of the photovoltaic parameters of the optimized OSC devices on the *u*FTEs before and after the *i*SPM under solar simulator AM 1.5G illumination. The OSCs on rigid ITO/glass is added as a reference.

Cathode		J_{SC}	V_{OC}	FF	PCE
P3HT:PC_{61}BM as a bulk heterojunction active layer					
*i*SPM	Avg.	-9.04	0.61	0.60	3.29
	Max.	-9.25	0.61	0.61	3.42
without	Avg.	-7.81	0.54	0.48	2.05
	Max.	-7.98	0.57	0.50	2.25
ITO/glass	Avg.	-9.18	0.60	0.60	3.30
	Max.	-9.40	0.60	0.61	3.46
D18-Cl:N3 as a bulk heterojunction active layer					
*i*SPM	Avg.	25.10	0.85	0.72	15.36
	Max.	25.55	0.86	0.75	16.51
without	Avg.	-23.21	0.83	0.66	12.83
	Max.	24.15	0.84	0.67	13.66
ITO/glass	Avg.	25.87	0.84	0.72	15.65
	Max.	25.92	0.85	0.75	16.70

Table S3. A summary of the averaged work function values over 10 samples of the pristine transparent ^{ZnO}NP films used in this work as measured by kelvin probe microscope and the work function values of ZnO reported by others.

Transparent metal-oxide-semiconductor	Work function (eV)	Refs.
^{ZnO}NPs	4.20	This work
ZnO	4.20	[5]
ZnO	4.10	[6]

Table S4. Work function values of the previously reported electrodes using ^{Ag}NWs and/or their composites with other transparent metal-oxide-semiconductor materials for comparing with the semi-integrated nanocomposite electrodes in cPI demonstrated in this work.

Electrode structure	Work function (eV)	Refs.
^{Ag}NWs	4.00	[7]
^{Ag}NWs:ZnO	4.54	[8]
^{Ag}NWs:AZO	4.31	[9]
^{Ag}NWs:^{ZnO}NPs	4.14	This work

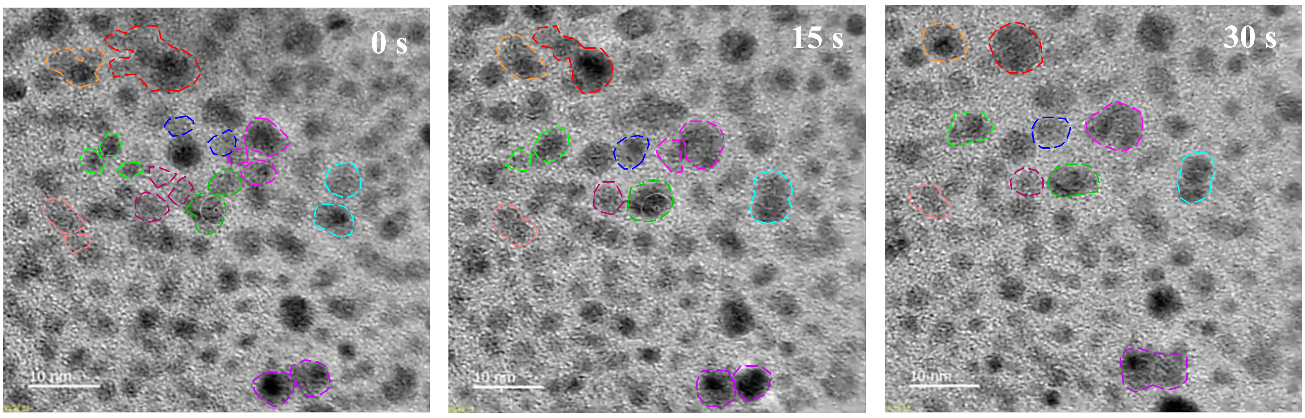

Figure S20 In-situ dynamics of multiple ^{ZnO}NPs. Sequential TEM images of in-situ dynamics of a number of ^{ZnO}NPs. The different evolving boundary is marked with different colors (red, green, blue, pink, etc., respectively). The time was arbitrarily set in the middle of the dynamics.

Influence of electron beam irradiation in TEM

It has been well reported that the relatively small-sized metal nanoparticles (a few nm in diameters) will sit in deep potential-energy wells by substrates of TEM and these NPs can get out of the wells by the respective critical amounts of energy [10]. Similarly, after the *i*SPM, the as-synthesized ^{ZnO}NPs with an average diameter of 5 nm dispersed on a copper grid substrate have been intensively identified as relaxing potential energy wells, allowing the initially trapped ^{ZnO}NPs to start to move and enabling their kinetics of the coalescence process to be captured by in-situ TEM observations. In the present work, the enforced current density was intentionally set as low as 3.99 e^5 electrons nm^{-2} s^{-1} equivalent to 6.8 A cm^{-2} to minimize the electron beam irradiation influence. It has been reported that the value is relatively lower and will elevate the temperature of ^{ZnO}NP less than 10 kelvin (*K*) [11], meaning that the influence of electron beam irradiation would be very limited. In contrast, the as-synthesized ^{ZnO}NPs before the *i*SPM were kept strongly trapped during the continuous electron beam irradiation from the TEM measurements of 5 minutes and no additional driving force was applied between them despite the adjacent ^{ZnO}NPs in close proximity (Figure S21). In fact, it has been well reported in many previous studies that surface diffusion can possibly be affected by electron beam irradiation [12-13] and observing the in-situ coalescence process of the small nanoparticles by TEM should be inevitably affected on the nanoparticles. The coalescence process may be accelerated by the irradiation such as charge excitations and/or increment of temperatures but that topic is beyond the scope of this work.

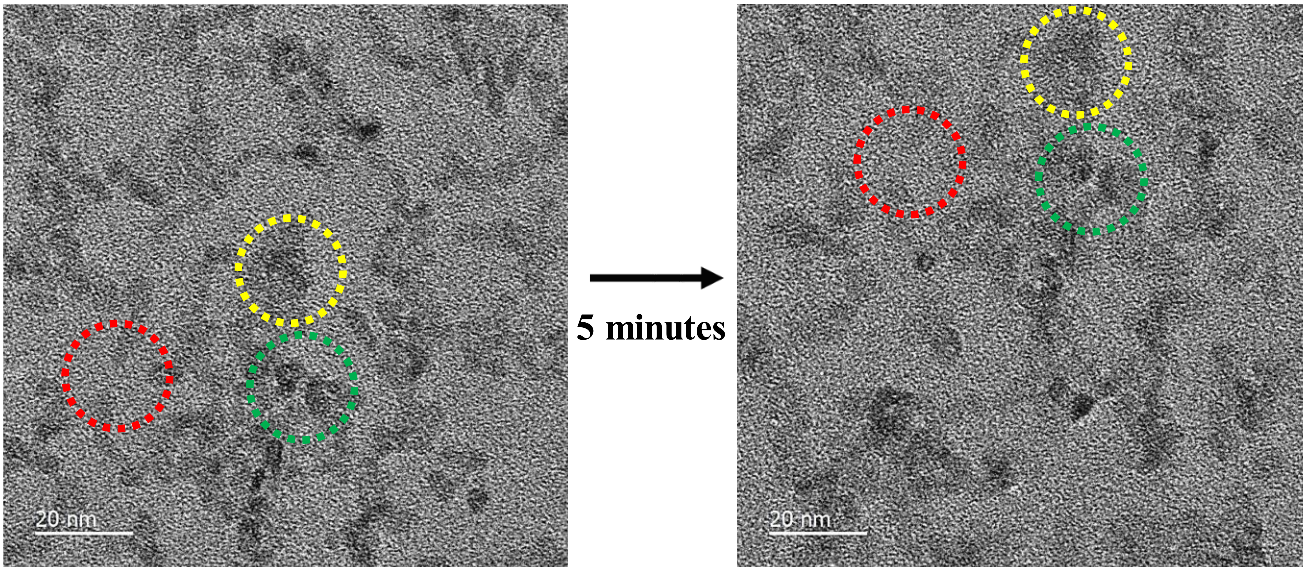

Figure S21 No morphological change of the ^{ZnO}NPs before the *i*SPM during the exposure to the same electron beam irradiation of the TEM. The sequential TEM images of the ^{ZnO}NPs before the *i*SPM during exposure to the same electron beam irradiation condition for 5 minutes. The circle with the same color is the same region after 5 minutes, in which the moved image was inevitably caused by the moving substrate. It can be concluded that there are no coalescence dynamics and merges among ^{ZnO}NPs found under the absence of the *i*SPM.

References

1. Swartz, E. T. Thermal boundary resistance. *Rev. Mod. Phys.* **61**, 605-668 (1989).
2. Ge, Y. *et al.* Direct Room Temperature Welding and Chemical Protection of Silver Nanowire Thin Films for High Performance Transparent Conductors. *J. Am. Chem. Soc.* **140**, 193–199 (2018).
3. Kim, J. *et al.* High Performance Flexible Transparent Electrode via One-Step Multifunctional Treatment for Ag Nanonetwork Composites Semi-Embedded in Low-Temperature-Processed Substrate for Highly Performed Organic Photovoltaics. *Adv. Energy Mater.* **10**, 1903919 (2020).
4. Pourjafari, D. *et al.* Functional Materials for Fabrication of Carbon-Based Perovskite Solar Cells: Ink Formulation and Its Effect on Solar Cell Performance. *Mater.* **16**, 3917 (2023).
5. Pourjafari, D. *et al.* Functional Materials for Fabrication of Carbon-Based Perovskite Solar Cells: Ink Formulation and Its Effect on Solar Cell Performance. *Mater.* **16**, 3917 (2023).
6. Noh, M. F. M. *et al.* The architecture of the electron transport layer for a perovskite solar cell. *J. Mater. Chem. C* **6**, 682-712 (2018).
7. Song, M. *et al.* Highly Efficient and Bendable Organic Solar Cells with Solution-Processed Silver Nanowire Electrodes. *Adv. Funct. Mater.* **23**, 4177-4184 (2013).
8. Zhao, X., Li, M., Jiang, L., Tang, H. & Guan, Y. Preparation of Device-Level ZnO-Covered Silver Nanowires Films and Their Applications as Sub-Electrode for Polymer Solar Cells. *Front. Chem.* **9**, 683728 (2021).
9. Chen, X. *et al.* Realizing Ultrahigh Mechanical Flexibility and >15% Efficiency of Flexible Organic Solar Cells via a “Welding” Flexible Transparent Electrode. *Adv. Mater.* **32**, 1908478 (2020).
10. Ajayan, P. M. & Marks, L. D. Experimental Evidence for Quasimelting in Small Particles. *Phys. Rev. Lett.* **63**, 279-282 (1989).
11. Cao, C. R. *et al.* Liquid-like behaviours of metallic glassy nanoparticles at room temperature. *Nat. Commun.* **10**, 1966 (2019).
12. Egerton, R. F. *Physical Principles of Electron Microscopy.* (Springer, 2005).
13. Surrey, A., Pohl, D., Schultz, L. & Rellinghaus, B. Quantitative Measurement of the Surface Self-Diffusion on Au Nanoparticles by Aberration-Corrected Transmission Electron Microscopy. *Nano Lett.* **12**, 6071-6077 (2012).
