# Supplementary material for: Tri-system integration in metal-oxide nanocomposites via in-situ solution-processed method for ultrathin flexible transparent electrodes
Source: Nat Commun. 2024 Mar 7;15:2070. doi: 10.1038/s41467-024-46243-6 (PMC10920808; doi:10.1038/s41467-024-46243-6)
Supplement: Supplementary file 3 — Description of Additional Supplementary Files [file 41467_2024_46243_MOESM3_ESM.pdf]

Description of Additional Supplementary Files

Supplementary Movie 1: In-situ ZnO NP-ZnO NP coalescence process

Supplementary Movie 2: In-situ liquid-like behaviors between Ag NW and ZnO NP

Supplementary Movie 3: Demonstration of folding test on flexible organic solar cells under the continuous AM 1.5G illumination
