# Supplementary material for: Tri-system integration in metal-oxide nanocomposites via in-situ solution-processed method for ultrathin flexible transparent electrodes
Source: Nat Commun. 2024 Mar 7;15:2070. doi: 10.1038/s41467-024-46243-6 (PMC10920808; doi:10.1038/s41467-024-46243-6)
Supplement: Supplementary file 2 — Peer Review File [file 41467_2024_46243_MOESM2_ESM.pdf]

nature portfolio

Peer Review File

Tri-system integration in metal-oxide nanocomposites via in-situ solution processed method for ultrathin flexible transparent electrodes

Open Access This file is licensed under a Creative Commons Attribution 4.0 International License, which permits use, sharing, adaptation, distribution and reproduction in any medium or format, as long as you give appropriate credit to the original author(s) and the source, provide a link to the Creative Commons license, and indicate if changes were made. In the cases where the authors are anonymous, such as is the case for the reports of anonymous peer reviewers, author attribution should be to 'Anonymous Referee' followed by a clear attribution to the source work. The images or other third party material in this file are included in the article's Creative Commons license, unless indicated otherwise in a credit line to the material. If material is not included in the article's Creative Commons license and your intended use is not permitted by statutory regulation or exceeds the permitted use, you will need to obtain permission directly from the copyright holder. To view a copy of this license, visit http://creativecommons.org/licenses/by/4.0/.

REVIEWER COMMENTS

Reviewer #1 (Remarks to the Author):

The manuscript titled "Tri-system integration in metal-oxide nanocomposites via in-situ solution processed method for ultrathin flexible transparent electrodes," authored by Kim et al., highlights the need for effective risk management to ensure the stability of ultrathin flexible transparent electrodes (uFTEs) under concurrent electrical bias and mechanical folding in high-humidity environments. The authors present compelling evidence of robust operational stability for solution-processed substrate-integrated uFTEs, achieved through the incorporation of Ag nanonets and ZnO nanoparticles. This robustness is attributed to the innovative in-situ solution processed method (iSPM), which facilitates a tri-system integration approach.

While the manuscript does exhibit certain commendable aspects within the domain of transparent conductors and could potentially be accepted in the high-impact journal Nat. Commun, it falls short of possessing enough impact in its current form, in my judgment. Addressing this limitation demands a major revision of the manuscript, alongside the incorporation of supplementary experimental work. My comments are listed below.

1. The authors convincingly demonstrate enhanced stability through the iSPM strategy, a critical facet for the dependable operation of practical devices. Nevertheless, an essential gap pertains to the exploration of the practical utility of these transparent electrodes within actual devices. Such integration mandates the evaluation of not only transparency and conductivity but also the pivotal roles played by topographic and surface electronic properties in determining overall device performance. To establish the pragmatic viability of the developed transparent electrode, the fabrication and comprehensive study of real devices are imperative. This constitutes my principal concern.

2. While the authors report an average reduction of 25% in resistance due to iSPM, there is an omission in the lack of error analysis for the resistance data. Including error margins is significant, as it not only enhances the credibility of the manuscript but also underscores the advantages linked to the outcomes, especially upon demonstrating the reliability and reproducibility of iSPM.

3. In addition to the employed AFM and TEM analyses, the manuscript would benefit from the incorporation of optical microscopic analyses. Such a comprehensive evaluation would encompass a broader perspective on the topographic properties of uFTEs, particularly on a macroscopic scale. The ensuing insights are pivotal in evaluating whether the iSPM engenders improved or deteriorated macroscopic properties in the conductive films, a fundamental consideration for successful integration into electronic devices.

4. While the manuscript presents stability data for uFTEs subjected to various folding cycles, it is crucial to also include stability data for uFTEs exposed to continuous bias current (without folding). This extra information can offer deeper insights into the degradation mechanism and the reasoning behind the improved stability after iSPM

5. Surface electronic properties should be examined using methods such as UPS or KPM, and the findings should be compared to those of other transparent conductors. This comparison will help establish the distinctiveness and benefits of the proposed approach.

Reviewer #2 (Remarks to the Author):

What are the noteworthy results?

A significant finding in this study pertains to the creation of a flexible transparent electrode characterized by robust electrical, mechanical, and thermal stability, along with resistance to humidity. This achievement was realized through the enhancement of surface adhesion at the heterogeneous interface between Silver Nanowires (AgNW) and Zinc Oxide Nanoparticles (ZnONP). To attain this objective, the author introduced an in-situ solution processing method (iSPM). Furthermore, this procedure unveiled an intriguing observation: the in-situ wetting of solid-state ZnONPs exhibited a quasi-liquid behavior as they infiltrated the AgNWs.

Will the work be of significance to the field and related fields? How does it compare to the established literature? If the work is not original, please provide relevant references.

The reviewer recognizes the significance of this study within the realm of flexible transparent electrodes. The notable achievement of enhancing surface adhesion at the heterogeneous interface, all without necessitating supplementary processes like post-annealing, bears substantial relevance in the arena of flexible transparent electrode investigations. Conventionally, established literature has often relied on techniques involving the application of additional energy, such as post-annealing or UV treatment. Nevertheless, it's crucial to note that these methods can potentially exert adverse effects on the polymer substrate serving as the foundational support material.

Does the work support your conclusions and claims, or is additional evidence needed?

The author's work substantiates the presented conclusions and claims. Nevertheless, some concerns arise with respect to thermal stability, uniform conductivity, and retention characteristics.

Firstly, regarding thermal stability, the authors assert it based on the heat dissipation mechanism depicted in Figure S6. However, it appears to imply indirect heat dispersion rather than direct thermal

stability. To address this, the authors should investigate how the transparent electrode's properties evolve when subjected to thermal stress, particularly focusing on changes in conductivity characteristics as a function of temperature applied to the substrate itself.

Secondly, the observed liquid-like behavior demonstrated in Figure 3, while advantageous for promoting adhesion, raises concerns about achieving uniform conductivity throughout the transparent electrode. The reviewer posits that this phenomenon may hinder the attainment of consistent sheet resistance or conductivity characteristics across the entire surface of the transparent electrode. The authors should thus consider and evaluate the uniformity of conductivity characteristics across the electrode's surface.

Thirdly, in relation to retention characteristics, the removal of polyvinylpyrrolidone (PVP) from the AgNW surface through the iSPM method may potentially increase the oxidation of AgNW over time. It is imperative for the authors to assess the long-term evolution of the transparent electrode's properties to ascertain the impact of PVP removal on its durability and stability.

Lastly, the reviewer recommends updating the references in your paper to include recent publications on ultrathin/flexible transparent electrodes with high Figure of Merit (FoM) values. Here are some examples of relevant papers published since 2021:

doi.org/10.1002/adom.202002010,

doi.org/10.1038/s41378-022-00353-7,

doi.org/10.1002/advs.202204746,

doi.org/10.1007/s40820-021-00735-y,

doi:10.1021/acsami.2c18738,

doi.org/10.1021/acsami.1c18866,

doi.org/10.1016/j.nanoen.2022.108028,

doi.org/10.1007/s40820-022-00859-9

Are there any flaws in the data analysis, interpretation and conclusions? Do these prohibit publication or require revision?

There are no flaws in the data division and interpretation conclusions of the paper and you don't see a need for a publication ban or revision.

Is the methodology sound? Does the work meet the expected standards in your field?

The methodology is sound and meets the standards.

Is there enough detail provided in the methods for the work to be reproduced?

Certainly, further elaboration is required on several aspects of the methodology:

1. Embedding Process: The authors should provide a detailed account of how they created the AgNW nanonets and ZnONP matrix semi-embedded in colorless polyimide (cPI).

2. Figure 1 Peeling-off Work: To enhance clarity for readers, a more comprehensive explanation of the peeling-off procedure depicted in Figure 1 is warranted. This should encompass the tools employed and the specific steps taken.

3. In the Methods section,

-Folding Task: In the Methods section, a comprehensive explanation of the folding procedure should be provided. This should encompass details about the equipment used for folding, including any specific devices or tools.

-Ethanol Solution: Specify the concentration (percentage) and grade of the ethanol solution utilized in the study, ensuring that readers can accurately replicate the experiment.

-NaBH4 Powder: Include pertinent information regarding the grade and source of NaBH4 powder employed in the research to facilitate reproducibility and transparency.

-Ultraviolet Ozone Treatment: Elaborate on the specifics of the ultraviolet ozone treatment, including the wavelength(s) of UV radiation applied and the duration of exposure.

-Mayer Bar Equipment: Describe the Mayer bar equipment and how it was utilized during the experimentation.

These additional explanations will ensure that other researchers can readily replicate the experimental procedures and better understand the intricacies of the methods employed in the study.

Reviewer #3 (Remarks to the Author):

This work reports "Tri-system integration in metal-oxide nanocomposites via in-situ solution processed method for ultrathin

flexible transparent electrodes". Silver nanowires were dispersed in zinc oxide nanoparticles matrix and the composites were further embedded in cPI to prepare ultrathin flexible transparent electrodes. The idea and preparation approach are quite similar to those reported in literature (Adv. Energy Mater. 2020, 10, 1903919) by the same group except the replacement of PEDOT:PSS by ZnO. Systematic study was conducted to reveal the liquid-like behaviors of transparent ZnO nanopartcles to Ag wire. The research topic is interesting. One important issue missing is a demonstration of the ultrathin flexible transparent electrodes for possible optoelectronic devices. The work may be accepted if the specific enquiries can be answered.

1.What is the purpose of developing such ultrathin flexible transparent electrode? A demostration in electronic device is suggested.

2.The preparation of Ag nanowire (Ag-NW) film by removing PVP with $NaBH_4$ was reported earlier by Duan et al. in JACS and in the presence of PEDOT:PSS by the same group,how about the reliability of this approaches?

3.Have the researchers observed similar liquid-like behaviours of other metal oxide nanoparticles to Ag-NW, so that this approach may be extended to other transparent electrodes?

4.On Page 8 of 18 line 284, please explain what is "ZnO (0001) interface"?

Point-by-point response to the referee's comments

Reviewer #1: The manuscript titled "Tri-system integration in metal-oxide nanocomposites via in-situ solution processed method for ultrathin flexible transparent electrodes," authored by Kim et al., highlights the need for effective risk management to ensure the stability of ultrathin flexible transparent electrodes (uFTEs) under concurrent electrical bias and mechanical folding in high-humidity environments. The authors present compelling evidence of robust operational stability for solution-processed substrate-integrated uFTEs, achieved through the incorporation of Ag nanonets and ZnO nanoparticles. This robustness is attributed to the innovative in-situ solution processed method (iSPM), which facilitates a tri-system integration approach.

While the manuscript does exhibit certain commendable aspects within the domain of transparent conductors and could potentially be accepted in the high-impact journal Nat. Commun, it falls short of possessing enough impact in its current form, in my judgment. Addressing this limitation demands a major revision of the manuscript, alongside the incorporation of supplementary experimental work. My comments are listed below.

Response: We greatly appreciate the reviewer's positive comments. We have addressed the comments of the reviewer accordingly as below.

Reviewer #1 Comment 1 and 5. The authors convincingly demonstrate enhanced stability through the iSPM strategy, a critical facet for the dependable operation of practical devices. Nevertheless, an essential gap pertains to the exploration of the practical utility of these transparent electrodes within actual devices. Such integration mandates the evaluation of not only transparency and conductivity but also the pivotal roles played by topographic and surface electronic properties in determining overall device performance. To establish the pragmatic viability of the developed transparent electrode, the fabrication and comprehensive study of real devices are imperative. This constitutes my principal concern. Surface electronic properties should be examined using methods such as UPS or KPM, and the findings should be compared to those of other transparent conductors. This comparison will help establish the distinctiveness and benefits of the proposed approach.

Response: We thank the reviewer for the comments. We have fabricated flexible organic solar cells (OSCs) on the *u*FTEs with a thickness of 10 μm and provided the current density-voltage (J-V) curves of the flexible OSCs using two different blended polymer active layers plotted in Figure S18a. The conventional rigid OSCs based on ITO/glass are also provided in Figure S18a for reference. Figure S18b shows the dark J-V characteristics of flexible OSCs using an active layer of D18-Cl:N3. The inset shows the SEM image of a cross-section of the flexible OSCs. Figure S18d describes the stability of flexible OSCs based on the *i*SPM-treated *u*FTE against the mechanical folding cycles of respective 1,000, 5,000, and 10,000 times with a folding radius of 1 mm under the continuous solar simulator AM 1.5G illumination. Figure S18e shows the evolution of the measured J_{SC}, V_{OC}, FF, and PCE from the flexible OSCs after the respective

folding cycles of Figure S18d (normalized to the initial value). Figure S18f exhibits the optical images of the flexible OSC device at the unfolding (left) and folding (right) cycles during the mechanical folding test with a folding radius of 1 mm under the solar simulator AM 1.5G illumination. We have added Table S2 with the comparison of the photovoltaic parameters of all the fabricated OSCs. Regarding the response to the comment 5, we have added the UPS spectra of the *u*FTEs in the cut-off regions as inserted in Figure S18c. The work functions of the *u*FTEs with the tri-system integration calculated from the UPS results have been compared to the other previously reported transparent conductors in Table S4. The KPM has been utilized for measuring the work functions of the ^{ZnO}NPs films in Table S3.

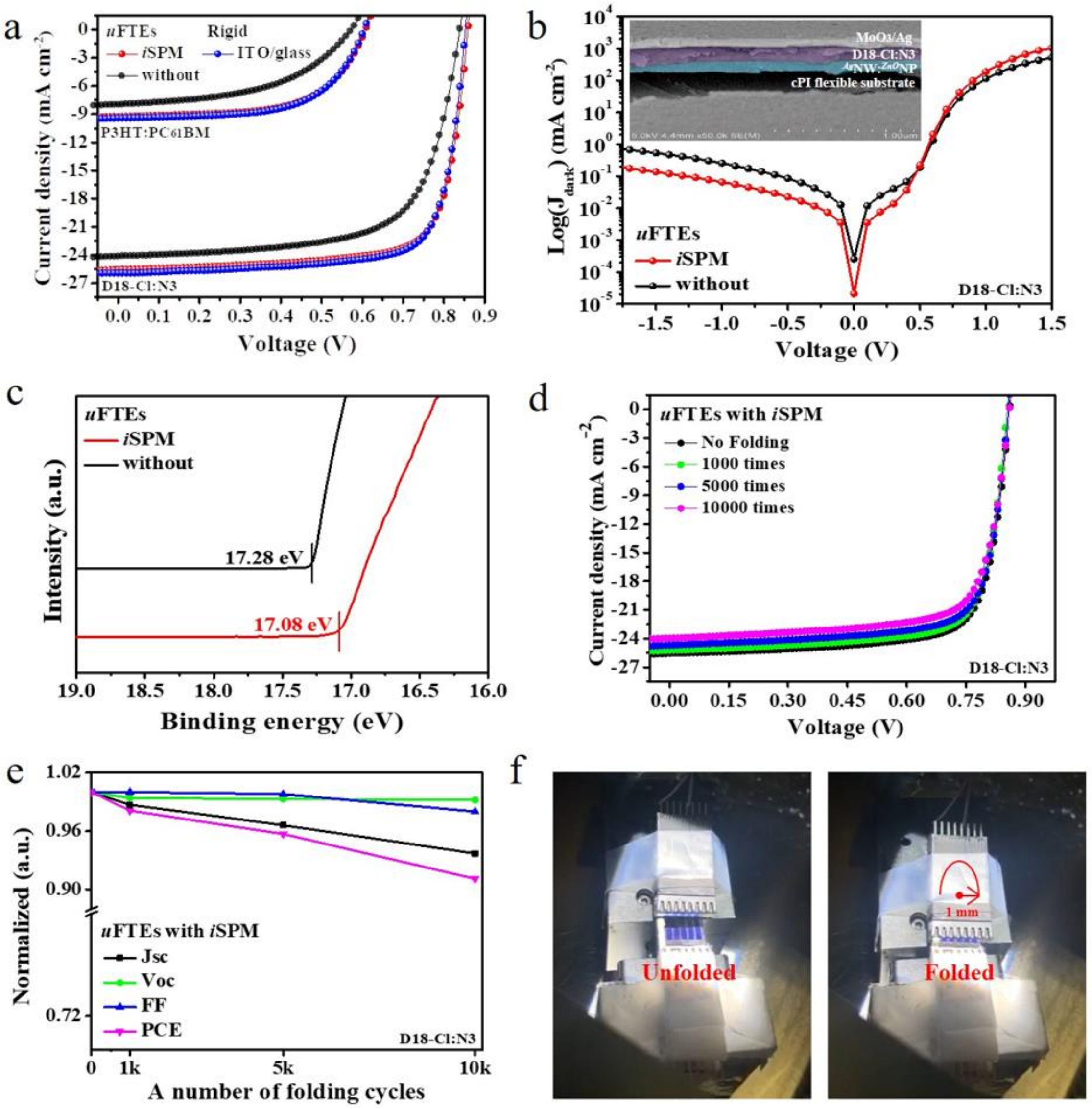

Figure S18 Characterization of the flexible OSCs on *u*FTEs with and without the *i*SPM. a, The J-V curves of the optimized flexible OSCs with P3HT:PC$_{61}$BM and D18-Cl:N3 as active layers under the AM 1.5G illumination. The J-V curves of the reference OSCs on rigid ITO/glass is also provided. **b**, The J-V curves under the dark state of the flexible OSCs with the D18-Cl:N3 active layer. The leakage current has been suppressed after the *i*SPM. In addition, the series resistance of the *i*SPM case is improved as displayed by the higher current density at a forward electrical bias over 0.5 V. The inset shows the SEM image of a cross-section of the optimized flexible OSCs with the *i*SPM. **c**, UPS spectra

of the *u*FTEs in the cut-off regions. The calculated work function value increases to 4.14 (with the *i*SPM) from 3.94 eV (without the *i*SPM). **d**, The evolved J-V curves of the flexible OSC based on the *i*SPM-treated *u*FTE during the test of respective 1,000, 5,000, and 10,000-time mechanical folding cycles under the continuous AM 1.5G illumination. The folding radius is 1 mm. **e**, The evolution of the normalized J_{SC}, V_{OC}, FF, and PCE measured from the J-V curves of Figure S18d for the respective mechanical folding cycles. **f**, Optical images of the flexible OSC device at the unfolding (left) and folding (right) cycles during the mechanical folding test with a folding radius of 1 mm under the AM 1.5G illumination. All the measurements were carried out in the glove box filled with a nitrogen environment.

Table S2. A summary of the photovoltaic parameters of the optimized OSC devices on the *u*FTEs before and after the *i*SPM under solar simulator AM 1.5G illumination. The OSCs on rigid ITO/glass is added as a reference.

Cathode		J_{SC}	V_{OC}	FF	PCE
P3HT:PC_{61}BM as a bulk heterojunction active layer					
*i*SPM	Avg.	-9.04	0.61	0.60	3.29
	Max.	-9.25	0.61	0.61	3.42
without	Avg.	-7.81	0.54	0.48	2.05
	Max.	-7.98	0.57	0.50	2.25
ITO/glass	Avg.	-9.18	0.60	0.60	3.30
	Max.	-9.40	0.60	0.61	3.46
D18-Cl:N3 as a bulk heterojunction active layer					
*i*SPM	Avg.	25.10	0.85	0.72	15.36
	Max.	25.55	0.86	0.75	16.51
without	Avg.	-23.21	0.83	0.66	12.83
	Max.	24.15	0.84	0.67	13.66
ITO/glass	Avg.	25.87	0.84	0.72	15.65
	Max.	25.92	0.85	0.75	16.70

Table S3. A summary of the averaged work function values over 10 samples of the pristine transparent ^{ZnO}NP films used in this work as measured by kelvin probe microscope and the work function values of ZnO reported by others.

Transparent metal-oxide-semiconductor	Work function (eV)	Refs.
^{ZnO}NPs	4.20	This work
ZnO	4.20	[5]
ZnO	4.10	[6]

Table S4. Work function values of the previously reported electrodes using ^{Ag}NWs and/or their composites with other transparent metal-oxide-semiconductor materials for comparing with the semi-integrated nanocomposite electrodes in cPI demonstrated in this work.

Electrode structure	Work function (eV)	Refs.
^{Ag}NWs	4.00	[7]
^{Ag}NWs:ZnO	4.54	[8]
^{Ag}NWs:AZO	4.31	[9]
^{Ag}NWs:^{ZnO}NPs	4.14	This work

The results and discussion are shown in page 20 of the Supporting Documents:

"**Performance and characterization of *u*FTEs in real optoelectronic devices**

To establish the pragmatic viability of the *u*FTEs, flexible organic solar cells (OSCs) fabricated on the *u*FTEs and their comprehensive study are presented in Figure S18. The *u*FTEs with a thickness of 10 μm were utilized due to the vulnerability of the extremely thin cPI (e.g. <0.5 μm) to chemical stability (e.g. dichlorobenzene, chloroform, etc.) and to ease the fabrication of OSCs. Figure S18a demonstrates that for OSCs with D18-Cl:N3 as the active layer the tri-system integration of the *u*FTEs improve the power conversion efficiency (PCE) from 13.66% (without the *i*SPM) to 16.51% which is comparable to that of 16.70 % based on rigid ITO/glass. The J_{SC}, V_{OC}, FF, and PCE are listed in Table S2. The improvement of the *i*SPM samples could be explained by three reasons. Firstly, it can be accessed by the suppressed leakage current (reverse bias regions of Figure S18b). Secondly, the series resistance reduces (forward bias regions after 0.5 V in Figure S18b). Lastly, it could be described by the modified surface work function value from 3.94 (without the *i*SPM) to 4.14 eV (with the *i*SPM) as calculated from the measured ultraviolet photoelectron spectroscopy (UPS) cut-off energy (Figure S18c). The small energy offset can be expected between the modified *u*FTEs and 4.20 eV of the work function of the bare ^{ZnO}NP film used as the electron transport layer of the OSC devices measured by kelvin probe microscope (KPM) (Table S3). As compared to the other reported ^{Ag}NW-based electrodes with different work functions (Table S4), the *u*FTEs with the tri-system integration could favor interface carrier transportation with a small energy offset for photovoltaic applications [4]. Notably, the PCE of the OSCs on the *u*FTEs with the *i*SPM has been preserved within 10 % after 10,000 times of folding cycles with a folding radius of 1 mm under the continuous AM 1.5G illumination (Figure S18d). The decreased PCE was closely related to the reduction of J_{SC} and FF while V_{OC} remained similar (Figure S18e). The video of the mechanical folding test of the OSC device conducted in the glovebox filled with nitrogen gas is provided (Video S3)".

We also added the following revisions to the content of the revised manuscript and Supporting Documents.

▪ Page 9 of the revised manuscript. "To establish the pragmatic viability of the developed *u*FTEs, the demonstration in real optoelectronic devices such as organic photovoltaics and its comprehensive study are provided in the Supporting Information (Figure S18). As a consequence, the unprecedentedly strong adhesion between ^{Ag}NWs and ^{ZnO}NPs has been experimentally demonstrated. The successfully semi-embedded nanocomposite electrode in the cPI matrix showed the extremely smooth surface roughness promoted by the tri-system integration, which played a decisive role in real optoelectronic devices".

▪ Page 11 of the revised manuscript. "The flexible OSCs on the developed *u*FTEs with and without the tri-system integration were fabricated and the maximum PCE of 13.66 % from the one without the *i*SPM has been substantially improved to that of 16.51 % for ones on the *i*SPM-modified *u*FTEs".

▪ Page 28 of Supporting Documents. "**3. Fabrication of flexible organic solar cell devices**

Two different pairs of donor and acceptor materials of P3HT:$PC_{61}BM$ and D18-Cl:N3 as organic active layers were purchased from Solarmer Materials Inc. 1,2-dichlorobenzene (DCB) anhydrous and chloroform (CF) ACS used for active layer solvents were purchased from Sigma-Aldrich. The solvent 1-butanol for ^{ZnO}NP solution as an electron transporting layer was bought from J&K Scientific LLC. Polydimethylsiloxane (PDMS) coated on a glass substrate was used as an adhesive film so that the peeled *u*FTEs were stuck to glass/PDMS as rigid supporting adhesive substrates for the flexible OSC device fabrication. All of the OSC devices with two different bulk heterojunction active layers were inverted devices with structures of cathode / ^{ZnO}NPs as electron transport layer / active layers / evaporated MoO_3 as hole transport layer / evaporated Ag as anode. The ITO-coated glass substrates of the reference OSCs were cleaned using sequential ultrasonication in deionized water, acetone, and ethanol for 15 minutes, respectively. Glass/ITO was treated by the UVO for 15 minutes. The ^{ZnO}NPs solution with a concentration of 15 mg mL^{-1} in 1-butanol as electron-transporting layers were deposited on both the *u*FTEs/PDMS/glass and ITO/glass by spin-coating at 3000 rpm for 40 s, followed by drying at 80 °C for 5 minutes. Two different kinds of organic bulk heterojunction active layers with P3HT:$PC_{61}BM$ (1:1, 20 mg mL^{-1} in DCB) and D18-Cl:N3 (1:1.2, 12 mg mL^{-1} in CF) were prepared. After transferring samples into the nitrogen-filled glovebox, these two organic active layers were spin-coated on the ^{ZnO}NPs layers, followed by post-treatment of 1 h slow growth and then annealing at 130 °C for 10 minutes for the P3HT:$PC_{61}BM$ film (static dispense at 670 rpm for 40 s, 30 uL), and annealing at 100 °C for 10 minutes for the D18-Cl:N3 film (dynamic dispense at 2500 rpm for 40 s, 17 uL). The devices were then finished by thermal evaporation of 7 nm MoO_3 and 100 nm Ag under a vacuum of 2×10^{-6} Torr in the high-vacuum deposition system with a device area of 0.08 cm^2".

▪ Page 28 of Supporting Documents. "The current density-voltage (J-V) characteristics of all the fabricated OSC devices were measured under a shadow mask with an opening area of 0.05 cm^2 using Enlitech AM 1.5 G solar simulator with a light intensity of 100 mW cm^{-2} calibrated with a standard silicon reference cell and the data was collected using a Keithley 2635 sourcemeter".

Reviewer #1 Comment 2. While the authors report an average reduction of 25% in resistance due to iSPM, there is an omission in the lack of error analysis for the resistance data. Including error margins is significant, as it not only enhances the credibility of the manuscript but also underscores the advantages linked to the outcomes, especially upon demonstrating the reliability and reproducibility of iSPM.

Response: We sincerely thank the reviewer for the comment. We have revised Figure S1a and Table S1 and added the error ranges in the sheet resistance of the ^{Ag}NWs networks and *u*FTEs after the *i*SPM.

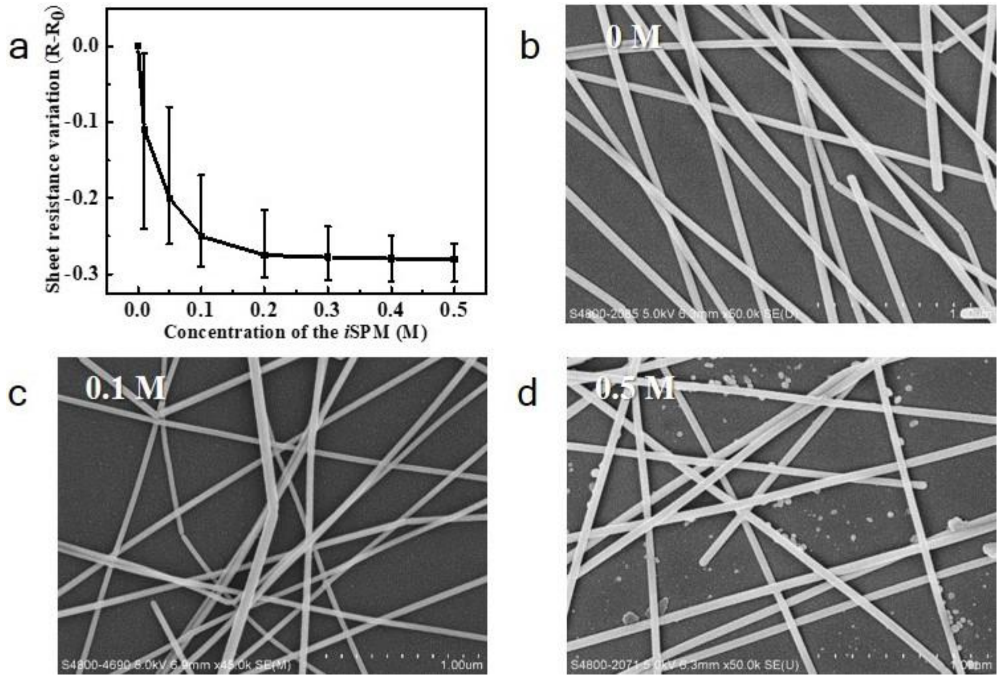

Figure S1 Ag nanonets before and after the *i*SPM with different concentrations of $NaBH_4$. In the process of the *i*SPM, the Ag nanonets placed on the supporting substrates had been dipped in the prepared $NaBH_4$ solution for 30 s. a, The averaged reduction of sheet resistance over 20 samples of Ag nanonets with the *i*SPM at different concentrations of $NaBH_4$ from 0 to 0.5 M. The rate of the reduction is almost saturated when $NaBH_4$ concentration is higher than 0.1 M. **b-d** SEM images of the Ag nanonets with the *i*SPM at different concentrations of 0, 0.1, and 0.5 M. **b**, 0 M. **c**, 0.1 M. **d**, 0.5 M. It was observed that there were many Ag nanoparticles produced by the highest concentration of 0.5 M likely due to the furious reduction reaction with Ag ions on the surface of ^{Ag}NWs. The optimized concentration for the *i*SPM was decided as 0.1 M with the consideration of the Ag debris production that there was no Ag debris together with decent sheet resistance reduction when it was 0.1 M.

Table S1 A summary of the average sheet resistance and diffused transmittance (stable over the wavelengths between 400 and 900 nm) over 20 samples of the composite electrode of Ag nanonets and ^{ZnO}NP matrix excluding the cPI substrate and the cPI substrate-integrated *u*FTEs before and after the *i*SPM, respectively.

*i*SPM	Before		After	
**Sample**	**Sheet resistance**	**Diffused transmittance**	**Sheet resistance**	**Diffused transmittance**
Nanocomposite	$10 \pm 1\ \Omega\ sq^{-1}$	92.9 %	$7.5 \pm 1\ \Omega\ sq^{-1}$	92.7 %
*u*FTEs	$10 \pm 1\ \Omega\ sq^{-1}$	88.6 %	$7.5 \pm 1\ \Omega\ sq^{-1}$	88.3 %

We also made the following changes in the content of the revised manuscript.

- Page 4. "The $NaBH_4$ concentration of 0.1 M has been decided for the optimized *i*SPM since it showed considerable reduction of sheet resistance from the original ones and the rate of the sheet resistance reduction was almost saturated after 0.1 M while there was almost no observed silver debris as a by-product (Figure S1)".

- Page 5. "The as-prepared nanocomposite electrodes without the cPI substrate and the cPI substrate-integrated *u*FTE showed an averaged diffused transmittance of >92 and >88 %, respectively. They showed negligible change in optical transparency at wavelengths between 400 nm and 900 nm with and without the *i*SPM (Figure S4), while the averaged reduction of 25 % in electrical sheet resistance to $7.5 \pm 1\ \Omega\ sq^{-1}$ for the *u*FTEs was confirmed after the *i*SPM (Table S1)".

Reviewer #1 Comment 3. In addition to the employed AFM and TEM analyses, the manuscript would benefit from the incorporation of optical microscopic analyses. Such a comprehensive evaluation would encompass a broader perspective on the topographic properties of uFTEs, particularly on a macroscopic scale. The ensuing insights are pivotal in evaluating whether the iSPM engenders improved or deteriorated macroscopic properties in the conductive films, a fundamental consideration for successful integration into electronic devices.

Response: We thank the reviewer for the comment. We have added Figure S17 with optical microscope images of the surface topography of the *u*FTEs for the comprehensive evaluation.

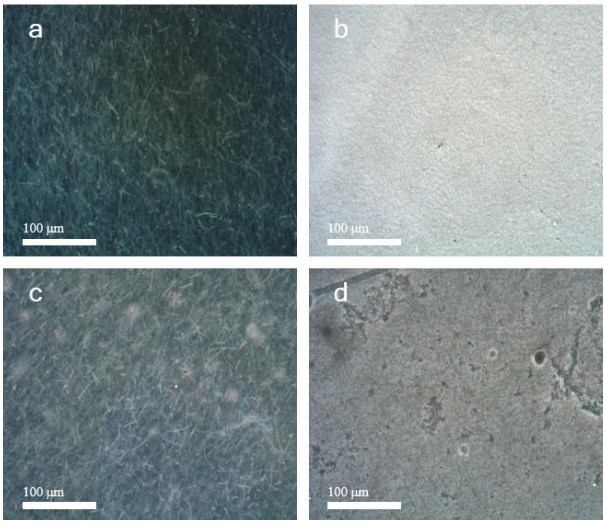

Figure S17 Optical microscopic images for surface topography of the substrate-integrated *u*FTEs. **a** and **c**, The dark mode for the *u*FTEs with and without the *i*SPM, respectively. **b** and **d**, The phase mode of the corresponding *u*FTEs. The poor coverage of ^{ZnO}NPs of the *u*FTEs without the *i*SPM can be seen (Figure S17d), which is similar results to the SEM image (Figure 2g) of the same *u*FTEs while the tri-system integration plays a decisive role in reducing the surface roughness of the *u*FTEs with the *i*SPM (Figure S17b), which is quite consistent with the SEM image (Figure 2h).

We also added the statement about the optical microscopic images for the surface topography of the *u*FTEs on Page 8 of the revised manuscript that "The optical microscopic images in dark and phase modes for surface topography of the substrate-integrated *u*FTEs are provided for comprehensive evaluation (Figure S17)".

Reviewer #1 Comment 4. While the manuscript presents stability data for uFTEs subjected to various folding cycles, it is crucial to also include stability data for uFTEs exposed to continuous bias current (without folding). This extra information can offer deeper insights into the degradation mechanism and the reasoning behind the improved stability after iSPM

Response: We thank the reviewer for the comment. We added Figure S8 with the stability results of the *u*FTEs exposed to continuous current density without mechanical stress/strain.

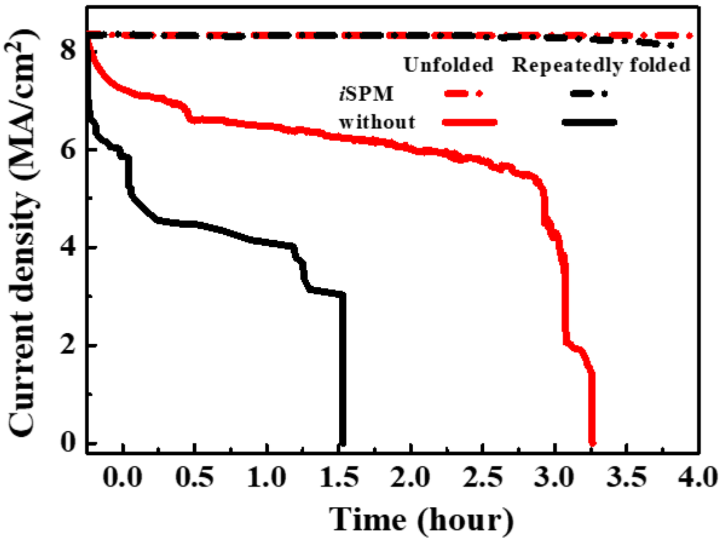

Figure S8 Real-time monitoring of the operational current density of the *u*FTEs with and without the *i*SPM under continuous current bias without mechanical folding. The measurement of the operational current density of the *u*FTEs with and without the *i*SPM under no mechanical stress/strain ("Unfolded" legend). The *u*FTEs were flattened by glass-supporting substrates and biased with the initial current density of 8.4 MA cm^{-2} for about 4 hours. For comparison, the results of Figure 1c were plotted together ("Repeatedly Folded" legend). Overall, between the samples without the *i*SPM, the repeatedly folded sample showed a relatively much shorter lifetime until they broke down as compared to the unfolded sample. On the other hand, the poor stability of the repeatedly folded sample has been significantly improved after the *i*SPM and showed similar results to the unfolded sample with the *i*SPM.

We also added the statement about the stability results of the *u*FTEs exposed to the continuous current density without mechanical stress/strain on Page 6 of the revised manuscript that "To provide different detailed results into the degradation, the *u*FTEs were tested under the continuous current bias without mechanical stress/strain as shown in Figure S8".

Reviewer #2:

What are the noteworthy results?

A significant finding in this study pertains to the creation of a flexible transparent electrode characterized by robust electrical, mechanical, and thermal stability, along with resistance to humidity. This achievement was realized through the enhancement of surface adhesion at the heterogeneous interface between Silver Nanowires (AgNW) and Zinc Oxide Nanoparticles (ZnONP). To attain this objective, the author introduced an in-situ solution processing method (iSPM). Furthermore, this procedure unveiled an intriguing observation: the in-situ wetting of solid-state ZnONPs exhibited a quasi-liquid behavior as they infiltrated the AgNWs.

Will the work be of significance to the field and related fields? How does it compare to the established literature? If the work is not original, please provide relevant references.

The reviewer recognizes the significance of this study within the realm of flexible transparent electrodes. The notable achievement of enhancing surface adhesion at the heterogeneous interface, all without necessitating supplementary processes like post-annealing, bears substantial relevance in the arena of flexible transparent electrode investigations. Conventionally, established literature has often relied on techniques involving the application of additional energy, such as post-annealing or UV treatment. Nevertheless, it's crucial to note that these methods can potentially exert adverse effects on the polymer substrate serving as the foundational support material.

Does the work support your conclusions and claims, or is additional evidence needed?

The author's work substantiates the presented conclusions and claims. Nevertheless, some concerns arise with respect to thermal stability, uniform conductivity, and retention characteristics.

Response: We greatly appreciate the reviewer’s positive comments and constructive suggestions. They are very valuable for revising and improving our manuscript.

Firstly, regarding thermal stability, the authors assert it based on the heat dissipation mechanism depicted in Figure S6. However, it appears to imply indirect heat dispersion rather than direct thermal stability. To address this, the authors should investigate how the transparent electrode's properties evolve when subjected to thermal stress, particularly focusing on changes in conductivity characteristics as a function of temperature applied to the substrate itself.

Response: We thank the reviewer for the comment and specific guidance. We added Figure S11 with the resistance evolution as the temperature applied to the substrates itself.

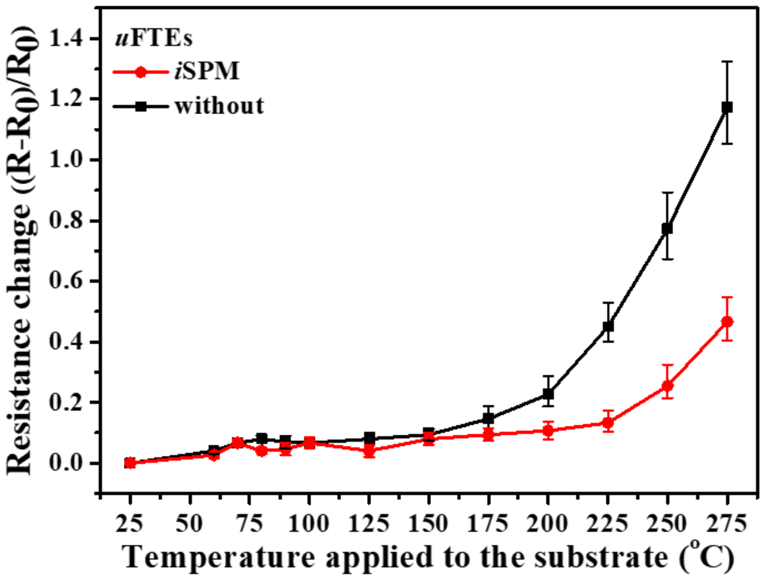

Figure S11 The change of resistance of the *u*FTEs with and without the *i*SPM with the temperature applied to the substrate. The resistance was directly measured from the *u*FTEs on a hot plate by using a multimeter. The resistance generally increased with the temperature. The *u*FTEs with the *i*SPM showed less resistance increment particularly when the temperature was higher than 150 ℃.

We also added the statement about the resistance evolution as the temperature applied to the substrate on Page 6 of the revised manuscript that "In addition, we investigated the direct thermal stress by measuring the resistance evolution in the *u*FTEs as a function of temperature of the substrate (Figure S11). It was found that the *i*SPM-modified *u*FTEs showed less resistance change with temperature particularly when the temperature was higher than 150 ℃ as compared to the unmodified ones".

Secondly, the observed liquid-like behavior demonstrated in Figure 3, while advantageous for promoting adhesion, raises concerns about achieving uniform conductivity throughout the transparent electrode. The reviewer posits that this phenomenon may hinder the attainment of consistent sheet resistance or conductivity characteristics across the entire surface of the transparent electrode. The authors should thus consider and evaluate the uniformity of conductivity characteristics across the electrode's surface.

Response: We thank the reviewer for the comment. We added Figure S5 with the uniformity of the *u*FTEs over the different surface regions.

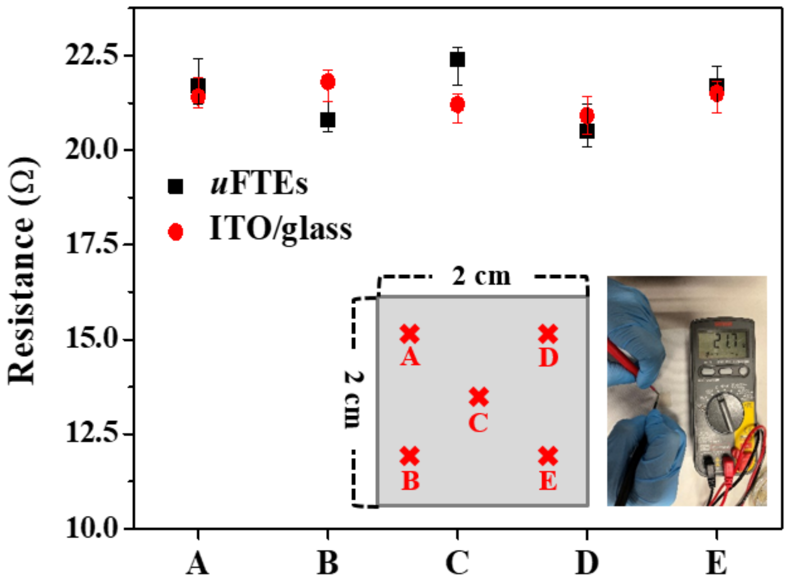

Figure S5 The resistance at different positions of the *u*FTEs with the *i*SPM. The ITO/glass is added as a comparison.

We also added the statement about the uniformity of the *u*FTEs on Page 5 of the revised manuscript that "It should be noted the *u*FTEs with the *i*SPM show very good resistance uniformity at different locations of the sample (Figure S5)".

Thirdly, It is imperative for the authors to assess the long-term evolution of the transparent electrode's properties to ascertain the impact of PVP removal on its durability and stability.

Response: We thank the reviewer for the comment. We added Figure S12a with the resistance stability of the ^{Ag}NWs-^{ZnO}NPs electrodes semi-embedded in cPI under air exposure for 35 days according to with and without PVP ligands through the *i*SPM.

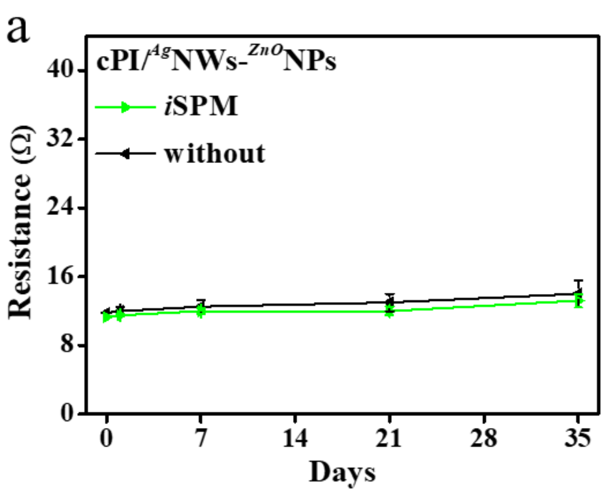

Figure S12 The resistance variation of cPI semi-embedded *u*FTEs under 35 days in air. a, The evolution of the resistance in cPI semi-embedded ^{Ag}NWs-^{ZnO}NPs electrodes according to with and without the *i*SPM. Generally, both the as-prepared ^{Ag}NWs passivated by the PVP ligands before the *i*SPM and the ^{Ag}NWs protected by the ^{ZnO}NP matrix after the *i*SPM showed stable resistance retention for 35 days of air exposure. It should be noted that the exposed areas of both the *u*FTEs to air are very narrow due to the semi-integration by the fluorinated cPI.

We also added the statement about the resistance stability under air exposure of 35 days on Page 6 of the revised manuscript that "Yet, there is a concern that after the PVP removals from the ^{Ag}NW networks, the air exposure could potentially be harmful. To confirm it, the *i*SPM-treated *u*FTEs with of PVP removal were placed in ambient environment for 35 days. Interestingly, the *u*FTEs with of PVP removal showed stable resistance which could be ascribed to both the passivation by the ^{ZnO}NPs matrix and fluorinated cPI despite the PVP removals (Figure S12a)".

Lastly, the reviewer recommends updating the references in your paper to include recent publications on ultrathin/flexible transparent electrodes with high Figure of Merit (FoM) values. Here are some examples of relevant papers published since 2021:

doi.org/10.1002/adom.202002010,

doi.org/10.1038/s41378-022-00353-7,

doi.org/10.1002/advs.202204746,

doi.org/10.1007/s40820-021-00735-y,

doi:10.1021/acsami.2c18738,

doi.org/10.1021/acsami.1c18866,

doi.org/10.1016/j.nanoen.2022.108028,

doi.org/10.1007/s40820-022-00859-9

Response: We thank the reviewer for the recommendation. We updated five papers among the suggested ones by numbering them 13-17 to the sentence on Page 2 of the revised manuscript that "The stringent requirements include a high figure of merit with well-balanced electrical and optical properties (i.e. <10 Ω sq.$^{-1}$ of sheet resistance and >90 % of diffused transmittance) and very smooth surface morphology (i.e. <1 nm of root-mean-square (RMS)) is preferred in terms of device applications [11-18].".

Are there any flaws in the data analysis, interpretation and conclusions? Do these prohibit publication or require revision?

There are no flaws in the data division and interpretation conclusions of the paper and you don't see a need for a publication ban or revision.

Is the methodology sound? Does the work meet the expected standards in your field?

The methodology is sound and meets the standards.

Is there enough detail provided in the methods for the work to be reproduced?

Certainly, further elaboration is required on several aspects of the methodology:

Response: We sincerely thank the reviewer for the positive comment and the valuable guidance on the methodology.

1. Embedding Process: The authors should provide a detailed account of how they created the AgNW nanonets and ZnONP matrix semi-embedded in colorless polyimide (cPI).

2. Figure 1 Peeling-off Work: To enhance clarity for readers, a more comprehensive explanation of the peeling-off procedure depicted in Figure 1 is warranted. This should encompass the tools employed and the specific steps taken.

Response: We sincerely thank the reviewer for the comment. We made significant changes to the Methods section and then they moved to the supporting documents.

▪ Page 27 of Supporting Documents. "

2. Fabrication of the entirely solution-processed *u*FTEs

Step 1: Preparation of the as-prepared ^{Ag}NWs-^{ZnO}NPs electrodes.

Rigid supporting substrates of 4 cm^2 with smooth surface roughness (e.g. silicon wafer, glass, etc.) were cleaned using deionized water, acetone, and ethanol. The cleaned substrates were then treated with ultraviolet-ozone (UVO) for 15 minutes. The ^{Ag}NWs networks were then formed on the treated substrates using Mayer rod coating or spin-coating. On top of the as-prepared ^{Ag}NW networks, the ^{ZnO}NPs was deposited by the same means.

Step 2: The *i*SPM on the as-prepared ^{Ag}NWs-^{ZnO}NPs electrodes.

The as-prepared ^{Ag}NWs-^{ZnO}NPs samples were treated by dipping them in the *i*SPM for 30 s. After that, the samples were washed with ethanol and deionized water. This *i*SPM process was repeated three times to get the final nanocomposite electrodes with the tri-system integration.

Step 3: Formation of cPI on *i*SPM-treated ^{Ag}NW-^{ZnO}NP samples.

On the *i*SPM-treated ^{Ag}NW-^{ZnO}NP samples, the PAA precursor solution of the cPI was coated to cover the whole surface by Mayer bar coating. Right after the PAA coating process, the samples will undergo the imidization processing by annealing with a gradual increase of temperature to 200 °C to form cPI. After the imidization processing temperatures, the samples were cooled down to room temperature.

Step 4: Peeling-off process

After cooling down to room temperature, the substrate-integrated ^{Ag}NW-^{ZnO}NP sample in the cPI were submerged in deionized water and then kept for the natural delamination for the formation of the electrode integrated with cPI substrate".

3. In the Methods section,

-Folding Task: In the Methods section, a comprehensive explanation of the folding procedure should be provided. This should encompass details about the equipment used for folding, including any specific devices or tools.

Response: We sincerely thank the reviewer for the comment. We made significant changes to the Methods section and then they moved to the supporting documents.

▪ Page 29 of Supporting Documents. "As for the folding task, we have used home-made folding machines with linear motion guides motorized with stepper motors. Regarding the software, we used the Arduino home-made program to adjust the linear distance with the minimum value of 1 mm. The detailed procedure of the folding task can be divided by 5 steps. Step (i): The *u*FTE samples were loaded to the sample holders with one end of the electrode fixed on the non-moving ground and the other end to the linear movement ground. Step (ii): For the measurement of the electrical properties, the *u*FTE samples were connected using the 3M electrical contact clips. Step (iii): The initial distance between the two ends of the electrode was set in the program to make the fixed *u*FTE sample straightened out. Step (iv): The moving distance and the number of forward/backward cycles were set in the program for running the folding test. Step (v): The *u*FTE samples were un-loaded after the folding test. A recorded video of the mechanical folding test of the OSC device is provided (Video S3)".

-Ethanol Solution: Specify the concentration (percentage) and grade of the ethanol solution utilized in the study, ensuring that readers can accurately replicate the experiment.

We revised to Ethanol (AnalaR NORMAPUR(R) ACS, ≥ 99.8%, VWR Chemicals).

-NaBH4 Powder: Include pertinent information regarding the grade and source of NaBH4 powder employed in the research to facilitate reproducibility and transparency.

We revised to $NaBH_4$ powder (ReagentPlus(R), 99%, Sigma-Aldrich).

-Ultraviolet Ozone Treatment: Elaborate on the specifics of the ultraviolet ozone treatment, including the wavelength(s) of UV radiation applied and the duration of exposure.

We revised to Ultraviolet-ozone (UVO) cleaner with a wavelength of 253.7 nm (Jelight Company, Inc.) and The cleaned substrates were then treated with ultraviolet-ozone (UVO) for 15 minutes.

-Mayer Bar Equipment: Describe the Mayer bar equipment and how it was utilized during the experimentation.

These additional explanations will ensure that other researchers can readily replicate the experimental procedures and better understand the intricacies of the methods employed in the study.

Response: We sincerely thank the reviewer for the comment. We made significant changes to the Methods section and then they moved to the supporting documents.

▪ Page 29 of Supporting Documents. "As for the Mayer bar equipment, the laboratory-type high-precision printing and coating machine with the built-in Mayer bar (from Peking University Yangtze Delta Institute of Optoelectronics) was used for the ^{Ag}NW, ^{ZnO}NP, and PAA coatings. The experimental adjustments have been carried out to both coating speed with 10-50 mm s^{-1} and height distance of less than 100 μm between the bar and the substrates depending on the target thickness and the viscosity of different materials/solutions".

Reviewer #3: This work reports "Tri-system integration in metal-oxide nanocomposites via in-situ solution processed method for ultrathin flexible transparent electrodes". Silver nanowires were dispersed in zinc oxide nanoparticles matrix and the composites were further embedded in cPI to prepare ultrathin flexible transparent electrodes. The idea and preparation approach are quite similar to those reported in literature (Adv. Energy Mater. 2020, 10, 1903919) by the same group except the replacement of PEDOT:PSS by ZnO. Systematic study was conducted to reveal the liquid-like behaviors of transparent ZnO nanopartcles to Ag wire. The research topic is interesting. One important issue missing is a demonstration of the ultrathin flexible transparent electrodes for possible optoelectronic devices. The work may be accepted if the specific enquiries can be answered.

Response: We greatly appreciate the reviewer's positive comments. We have addressed the comments of the reviewer accordingly as below.

Reviewer #3 Comment 1. What is the purpose of developing such ultrathin flexible transparent electrode? A demostration in electronic device is suggested.

Response: We thank the reviewer for the comment. There are many advantages in terms of ultrathin, flexible, and transparent properties of *u*FTEs. For instance, the "flexible" electrodes are an indispensable component in flexible electronics for advancing their applications. They make higher levels of innovation and designs in flexible electronics. Besides, compared with rigid device manufacturing, the ultimate low-cost manufacturing can be realized such as a roll-to-roll imprinting process with inexpensive plastic substrates. Furthermore, the "ultrathin" electrodes can form an intimate contact with arbitrary shapes such as human skin and implantable system without imposing constraints due to mechanical force or weight. Such favorable features are critical for reliable monitoring key biomedical and physiological information and effective treatment and management of specific chronic diseases. Finally, the "transparency" of the electrodes can preserve visual information to the electronic devices for comprehensive optical analysis and applications. For example, transparency can offer optical imperceptibility, alleviating reluctance to wear the optoelectronic devices on skin of human and animals. There are a variety of applications for such an indispensable *u*FTEs such as health care and monitoring, thin-film heaters, temperature and infrared sensors, display, and organic solar cells [*Nature* **499**, 458–463 (2013), *Acc. Chem. Res.* **52**, 523–533 (2019), and *Chem. Rev.* **123**, 9982-10078 (2023)]. Among them, we have fabricated flexible organic solar cells (OSCs) based on the *u*FTEs as shown in Figure I. The preliminary power conversion efficiency (PCE) of 16.51% was achieved, which is comparable to that of 16.70 % based on rigid ITO/glass as a reference. The device efficiency could be further improved after the optimizing the device structure. All the important OSC parameters of J_{SC}, V_{OC}, FF, and PCE are listed in Table I.

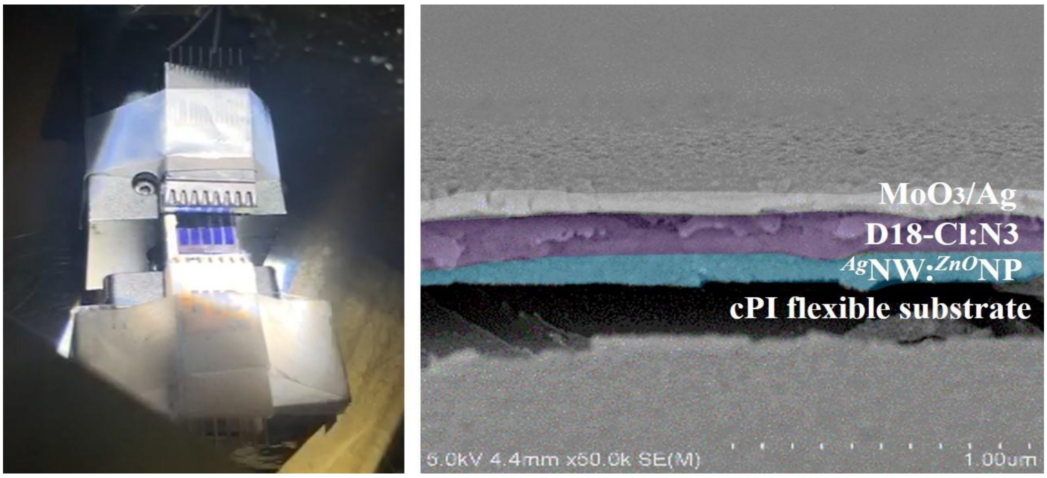

Figure I An optical image of the flexible OSC device producing electricity under the AM 1.5G illuminated state (left) and a cross-sectional SEM image of the flexible OSC device (right). The flexible OSC devices have structures of cPI as flexible substrate / ^{Ag}NWs-^{ZnO}NPs as cathode / ^{ZnO}NPs as electron transport layer / D18-Cl:N3 as active layer / evaporated MoO3 as hole transport layer / evaporated Ag as anode.

Table I A summary of the photovoltaic parameters of flexible OSC devices based on the *u*FTEs under solar simulator AM 1.5G illumination. The rigid conventional ITO/glass is added for reference.

Cathode		J_{SC}	V_{OC}	FF	PCE
D18-Cl:N3 as a bulk heterojunction active layer					
Flexible OSC devices	Avg.	25.10	0.85	0.72	15.36
	Max.	25.55	0.86	0.75	16.51
ITO/glass references	Avg.	25.87	0.84	0.72	15.65
	Max.	25.92	0.85	0.75	16.70

Reviewer #3 Comment 2. The preparation of Ag nanowire (Ag-NW) film by removing PVP with NaBH4 was reported earlier by Duan et al. in JACS and in the presence of PEDOT:PSS by the same group,how about the reliability of this approaches?

Response: We thank the reviewer for the comment. We have added Figure S12b with the resistance stability of previously reported cPI semi-embedded ^{Ag}NWs and ^{Ag}NWs-PEDOT:PSS electrodes under air exposure for 35 days after the PVP-removal from the ^{Ag}NW surface, and the comparison of the PVP-removed ^{Ag}NWs-^{ZnO}NPs of this work.

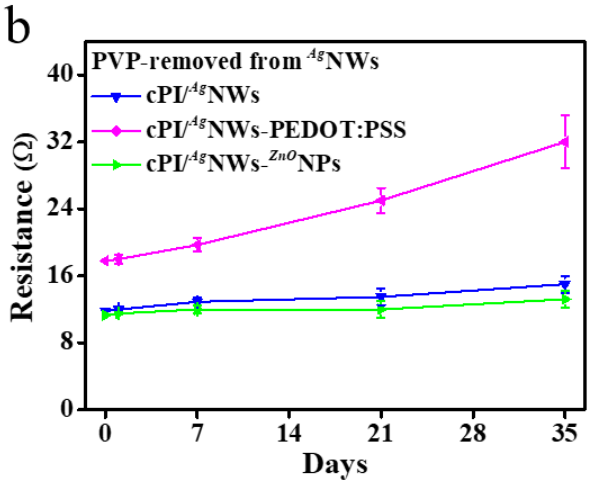

Figure S12 The resistance variation of cPI semi-embedded *u*FTEs under 35 days in air. b, The resistance of both the previously reported ^{Ag}NWs and ^{Ag}NW-PEDOT:PSS electrodes semi-embedded in cPI after the PVP-removal from the ^{Ag}NW surface was measured and compared to the PVP-removed ^{Ag}NW-^{ZnO}NP electrode of this work. The PVP-removals for the bare ^{Ag}NWs and ^{Ag}NWs-PEDOT:PSS were prepared according to reference [2] and [3] respectively. Overall, the PVP-removed ^{Ag}NWs showed stable resistance retention properties, which could be ascribed to the protection of semi-embedding in cPI while the ^{Ag}NW-^{ZnO}NP of this work showed a better protection through the coverage of ^{ZnO}NP matrix to ^{Ag}NWs. However, the retention resistance properties of the composite electrode of ^{Ag}NWs-PEDOT:PSS exhibited gradual degradation during the test period of 35 days due to the acidic properties of PEDOT:PSS.

Reviewer #3 Comment 3. Have the researchers observed similar liquid-like behaviours of other metal oxide nanoparticles to Ag-NW, so that this approach may be extended to other transparent electrodes?

Response: In preliminary results, different types of transparent metal-oxide-semiconductor nanoparticles (^{TMOS}NPs) such as titanium oxide (SnO_2), tin oxide (TiO_x), and nickel oxide (NiO_x) have been incorporated to ^{Ag}NWs by using the same *i*SPM approach. Interestingly, we observed the liquid-like behaviors by doing detailed TEM analysis among these compositions. However, the outputs have not been optimized yet. Thus, we only show the parts of the undergoing characterization in Figure II about the optical transparency and operational stability against the multi-loading tests. The progress will be reported in future works.

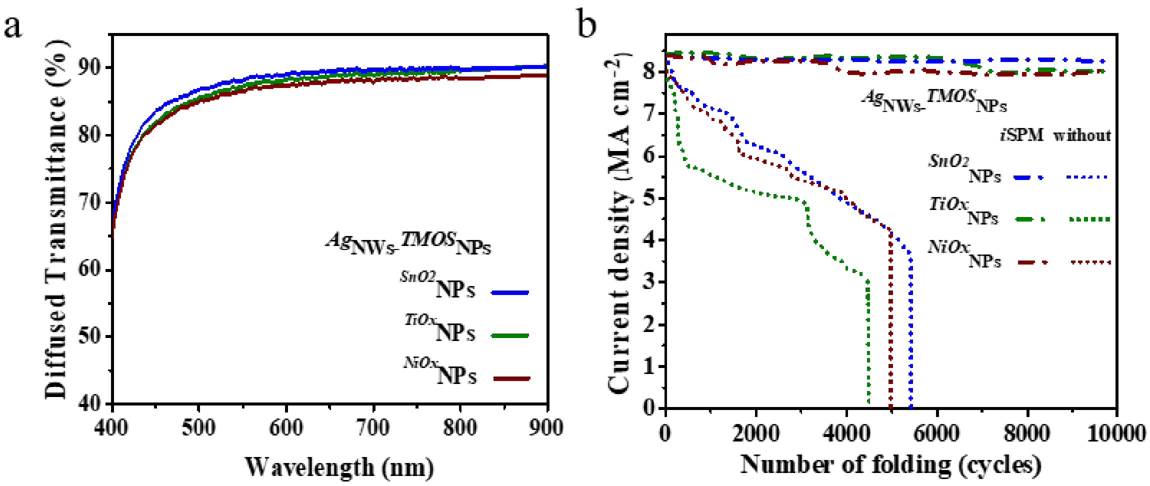

Figure II Preliminary characterization of the *u*FTEs made of ^{Ag}**NWs with different transparent metal-oxide-semiconductor nanoparticles (**^{TMOS}**NPs) including** SnO_2**,** TiO_x**, and** NiO_x**. a**, The optical transmission of the cPI integrated *u*FTEs. **b**, The current density of the *u*FTEs with and without the *i*SPM under multi-loading test of folding characterization with an initial 8.4 MA cm^{-2} and folding radius of 1 mm, and the simultaneous exposure to a high humid condition of relative humidity 85 %.

Reviewer #3 Comment 4. On Page 8 of 18 line 284, please explain what is "ZnO (0001) interface"?

Response: We thank reviewer for the comment. We have revised to "ZnO (0001) surface" on Page 8 of the revised manuscript.

REVIEWER COMMENTS

Reviewer #1 (Remarks to the Author):

The authors have addressed all my concerns, and I recommend the manuscript for publication.

Reviewer #2 (Remarks to the Author):

1. Thermal Safety:

While acknowledging the author's effort to design a comprehensive experimental plan to evaluate thermal stability, a key concern emerges regarding the resilience of the ultrathin film, incorporating nanowires within a polyimide structure, under the specified conditions. Notably, polyimide materials typically exhibit limited thermal endurance, and the reported capability to withstand temperatures as high as 275 degrees Celsius raises questions about the material's structural integrity. Considering the intrinsic properties of polyimide materials, it is anticipated that exposure to elevated temperatures, particularly on a hot plate as opposed to an oven, may result in heightened vulnerability and potential damage. Given the unconventional nature of this thermal tolerance, additional details on the experimental design, protective measures implemented to prevent potential damage or distortion, and any available data substantiating the film's stability within the elevated temperature range are deemed critical for verification. This request for supplementary information stems from a foundational need to reconcile the observed thermal resilience with established expectations for polyimide materials. Clarification in this regard is paramount to establishing confidence in the reliability of the reported data. Elaboration on the experimental setup, procedures, and any measures taken to prevent damage or deformation of the film is requested. Additionally, additional data points within the temperature range of 50 to 275 degrees Celsius would enhance comprehension of the film's response across this spectrum.

2. Uniformity of Conductivity:

It has been observed that resistance measurements were conducted by making contact with two probe tips on the surface of the transparent electrode. However, it is crucial to emphasize that transparent electrodes necessitate the quantification of sheet resistance rather than line resistance. To ensure accurate representation and interpretation of conductivity characteristics, future assessments should employ methodologies specifically designed for capturing sheet resistance, thereby providing a more comprehensive understanding of the uniformity and performance of the transparent electrode.

To bolster the clarity of the data, it is suggested that information be provided on the thickness of both the Indium Tin Oxide (ITO) film and the ultrathin Flexible Transparent Electrodes (uFTEs). This additional

detail will contribute to a comprehensive understanding of the materials employed and their respective roles in the study.

3. Durability and Stability:

The evaluation of durability and stability is acknowledged as appropriate, providing confidence in the robustness of the study's findings.

4. References:

The inclusion of additional references is deemed appropriate, contributing to the enrichment of the literature review and aligning the study with recent developments in the field.

5. Additional Required Data:

The incorporation of supplementary data is acknowledged as appropriate and commendable, reinforcing the completeness of the research presentation.

These suggested modifications aim to enhance the overall clarity and completeness of the manuscript, ensuring a comprehensive understanding of the experimental procedures and findings.

Reviewer #3 (Remarks to the Author):

The manuscript has been carefully revised according to reviewers' comments. I would like to suggest its acceptance.

Point-by-point response to the 2nd referee's comments

Reviewer #2 Comment 1. Thermal Safety: While acknowledging the author's effort to design a comprehensive experimental plan to evaluate thermal stability, a key concern emerges regarding the resilience of the ultrathin film, incorporating nanowires within a polyimide structure, under the specified conditions. Notably, polyimide materials typically exhibit limited thermal endurance, and the reported capability to withstand temperatures as high as 275 degrees Celsius raises questions about the material's structural integrity. Considering the intrinsic properties of polyimide materials, it is anticipated that exposure to elevated temperatures, particularly on a hot plate as opposed to an oven, may result in heightened vulnerability and potential damage. Given the unconventional nature of this thermal tolerance, additional details on the experimental design, protective measures implemented to prevent potential damage or distortion, and any available data substantiating the film's stability within the elevated temperature range are deemed critical for verification. This request for supplementary information stems from a foundational need to reconcile the observed thermal resilience with established expectations for polyimide materials. Clarification in this regard is paramount to establishing confidence in the reliability of the reported data. Elaboration on the experimental setup, procedures, and any measures taken to prevent damage or deformation of the film is requested. Additionally, additional data points within the temperature range of 50 to 275 degrees Celsius would enhance comprehension of the film's response across this spectrum.

Response: We thank the reviewer for this valuable comment. Polyimides have been of great technological importance in microelectronics due to their high-temperature stability [1-2]. The strong polymer chain interactions make polyimides very high glass transition temperatures (T_g) over 300 °C and some of them even approaches 400 °C [3-5]. These high T_g values indicate that the polymer chains at the molecular level significantly restrict their movement, resulting in the excellent thermal stability. The T_g over 300°C for our studied polyimide has been previously reported [6]. As shown in Figure S12, our polyimide showed no obvious physical damage, distortion, or deformation even after exposure to elevated temperatures up to 275 °C on a hot plate.

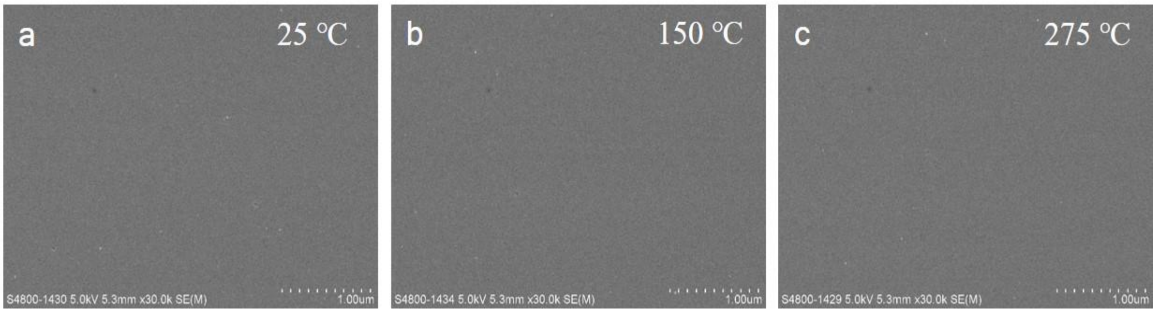

Figure S12 The surface morphology evolution of cPI after exposure to different temperatures on a hot plate. The SEM images after exposure to **a**, room temperature of 25 °C. **b**, 150 °C. **c**, 275 °C. No trace of damage, distortion, or deformation are observed after the thermal test with temperatures as high as 275 °C. The cPI flexible substrates were tested by directly placing on a hot plate at the designed temperatures for 30 minutes.

We have performed additional electrical measurements at temperatures between 50 to 275 °C using the same hot plate as shown in Figure S11. The resistance generally increased with the temperature [7]. The *i*SPM treated *u*FTEs showed lower resistance, particularly when the temperature of the hot plate was higher than 150 °C.

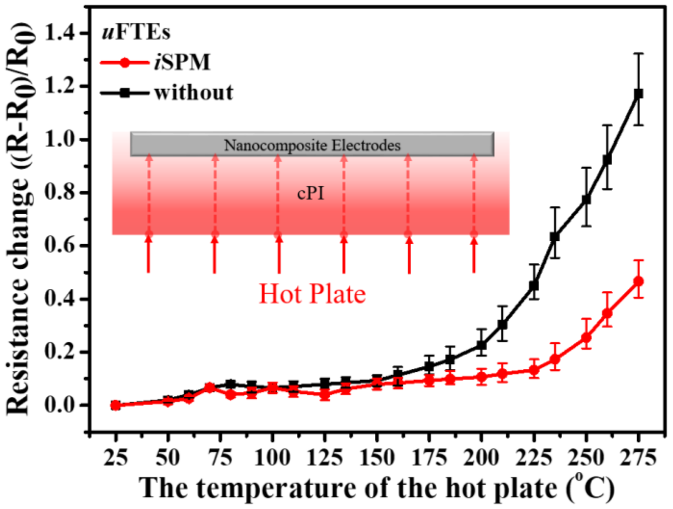

Figure S11 The ratio of resistance changes of the *u*FTEs with and without the *i*SPM against the temperature applied to the cPI substrate. The resistance was directly measured from the *u*FTEs when the samples were put a hot plate at elevated temperatures for 5 minutes.

We also added the statement about the robust thermal endurance of cPI on Page 6 of the revised manuscript that "In addition, we investigated the thermal stress on both *u*FTEs and cPI by characterizing the resistance changes of the *u*FTEs and the SEM images for surface morphology evolution of cPI substrates with the elevated temperatures, respectively (Figure S11 and S12)".

Reviewer #2 Comment 2. Uniformity of Conductivity: It has been observed that resistance measurements were conducted by making contact with two probe tips on the surface of the transparent electrode. However, it is crucial to emphasize that transparent electrodes necessitate the quantification of sheet resistance rather than line resistance. To ensure accurate representation and interpretation of conductivity characteristics, future assessments should employ methodologies specifically designed for capturing sheet resistance, thereby providing a more comprehensive understanding of the uniformity and performance of the transparent electrode. To bolster the clarity of the data, it is suggested that information be provided on the thickness of both the Indium Tin Oxide (ITO) film and the ultrathin Flexible Transparent Electrodes (uFTEs). This additional detail will contribute to a comprehensive understanding of the materials employed and their respective roles in the study.

Response: Thank you for valuable comments from the reviewer which can help to improve quality of our work. We have measured the sheet resistance (Ω sq^{-1}) by a four-point probe to characterize the uniformity of electrode conductivity of *u*FTEs and commercial ITO/glass as

shown Figure S5. The results show that the conductivity is uniform for both electrodes. The measured thickness of ^{Ag}NW-^{ZnO}NP film in *u*FTE and the ITO film are 60 and 150 nm, respectively. Notably, the conductivity of *u*FTEs with a thinner thickness is lower to that of the thick ITO/glass. Furthermore, the flexibility of *u*FTEs makes them more attractive in state-of-the-art research fields and modern device innovations such as wearable optoelectronic devices and implantable bioelectronics.

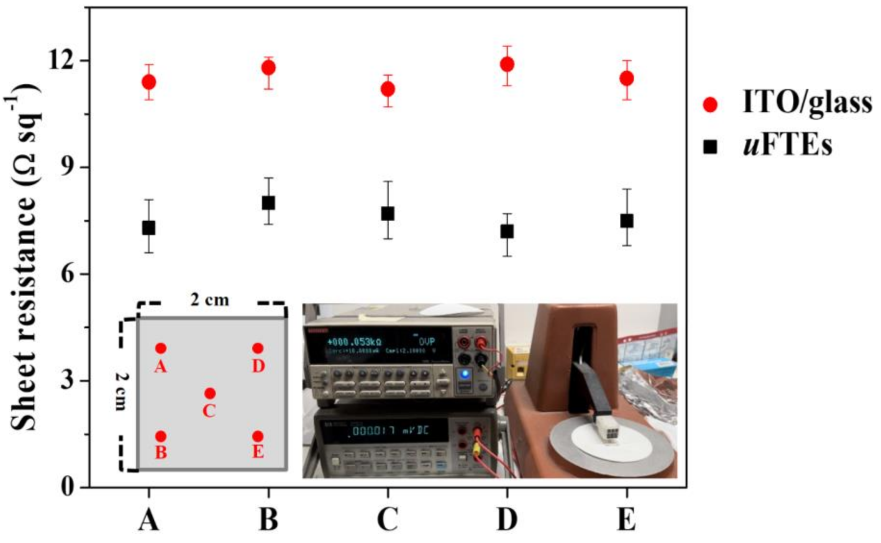

Figure S5 The sheet resistance uniformity of the *u*FTEs. The sheet resistance uniformity at different positions of the *i*SPM treated *u*FTEs measured by a four-point probe. The ITO/glass is added as a comparison. The thickness of commercial ITO film on a glass substrate and the conductive ^{Ag}NW-^{ZnO}NP film in *u*FTE is about 150 and 60 nm, respectively.

Reviewer #2 Comment 3, 4, and 5.

3. Durability and Stability: The evaluation of durability and stability is acknowledged as appropriate, providing confidence in the robustness of the study's findings.

4. References: The inclusion of additional references is deemed appropriate, contributing to the enrichment of the literature review and aligning the study with recent developments in the field.

5. Additional Required Data: The incorporation of supplementary data is acknowledged as appropriate and commendable, reinforcing the completeness of the research presentation.

These suggested modifications aim to enhance the overall clarity and completeness of the manuscript, ensuring a comprehensive understanding of the experimental procedures and findings.

Response: We greatly appreciate the reviewer time and effort that have dedicated to providing comprehensive results in our work. We are truly honored to have the reviewer's insightful comments on our paper.

Reference

[1] Wilson, D., Stenzenberger, H. D. & Hergenrother, P. M. Ed., "Polyimides", Chapman and Hall, New York, N.Y. (1990).

[2] Ghosh, M. K. & Mittal, K. L. Ed., "Polyimides: Fundamentals and Applications," Marcel Dekker, Inc., New York, N.Y. (1996).

[3] Kang, H. A., Chung, I. S., Kakimoto, M. & Kim, S. Y. Synthesis and Characterization of Polyimides from Unsymmetrical Diamine with Cyano Groups. *Polym. J.* **33**, 284-289 (2001).

[4] Fang, Y. *et al.* Terphenyl-based colorless and heat-resistant polyimides with a controlled molecular structure using methyl side groups. *Polym. Chem.* **13**, 5105-5115 (2022).

[5] Li, D. *et al.* Synthesis of colorless polyimides with high T_g and low coefficient of thermal expansion from benzimidazole diamine containing biamide. *J. Polym. Sci.* **61**, 818-828 (2023).

[6] Jiang, P. *et al.* The Influences of Sulfoxide Electron Traps in Transparent Polyimides with Low Retardation, Yellow Index, and CTE. *Macromol. Mater. Eng.* **306**, 2000606 (2021).

[7] Zhao, X., Li, M., Jiang, L., Tang, H. & Guan, Y. Preparation of Device-Level ZnO-Covered Silver Nanowires Films and Their Applications as Sub-Electrode for Polymer Solar Cells. *Front. Chem.* **9**, 683728 (2021).
